# Organorhodium and Iridium-Containing Derivatives of the 48-Tungsto-8-phosphate Wheel: Synthesis, Characterization, and Catalytic Activity

**DOI:** 10.1021/acs.inorgchem.5c06046

**Published:** 2026-03-18

**Authors:** Ali S. Mougharbel, Saurav Bhattacharya, Anupam Sarkar, Anton-Jan Bons, Tom D’hondt, Helge Jaensch, Ulrich Kortz

**Affiliations:** † School of Science, 84498 Constructor University, Campus Ring 1, 28759 Bremen, Germany; ‡ Department of Chemistry, Birla Institute of Technology and Science Pilani, KK Birla Goa Campus, Zuarinagar, 403726 Sancoale, Goa, India; § ExxonMobil Petroleum & Chemical, BV 1831 Machelen, Belgium

## Abstract

We report on the synthesis, characterization, and catalysis of organorhodium- and iridium-containing derivatives of the 48-tungsto-8-phosphate wheel, [{Rh­(Cp*)­(H_2_O)}_4_P_8_W_48_O_184_]^32–^ (**1**) and [{Ir­(Cp*)­(H_2_O)}_4_P_8_W_48_O_184_]^32–^ (**2**). The novel polyanions **1** and **2** were synthesized by the reaction of (MCp*Cl_2_)_2_ (M = Rh^III^, Ir^III^) with the mixed potassium–lithium salt of the cyclic P_8_W_48_ precursor in an aqueous medium using mild one-pot conditions, and four organometallic moieties are covalently bound to the central cavity of the P_8_W_48_ wheel via two M-O­(W) oxygen bridges. Both polyanions were structurally characterized in the solid state by single-crystal X-ray diffraction, FT-IR spectroscopy, and thermogravimetric analysis, as well as in solution by ^31^P and ^13^C NMR spectroscopy. The hydrogenation of olefins was investigated after supporting polyanions **1** and **2** on mesoporous SBA15. The supported catalysts exhibited high activity in the selective hydrogenation of o-xylene with very little cracking products, even under high temperature and pressure conditions.

## Graphical abstract

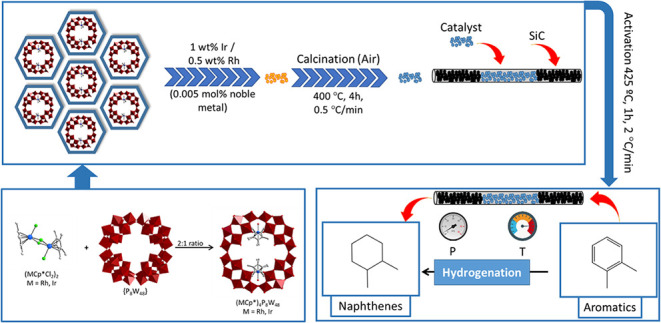

## Introduction

Noble metal-based catalysts are of great importance in the chemical and petrochemical industries. Several industrial processes use platinum- or palladium-based catalysts for the hydrogenation of acetylene in ethylene mixtures, hydrocracking processes, and hexamethylenediamine or cyclohexanone synthesis. In addition, one of the largest markets for noble metal catalysts is the automotive industry, where noble metals such as platinum, palladium, and rhodium are the main components in catalytic converters of cars.^1^ Immobilizing nanoparticles on the surface of suitable support materials is a well-established strategy to properly disperse the active sites in order to increase activity.^2^ However, under reaction conditions or during catalyst regeneration to remove coke and poisons, the nanoparticles tend to move and agglomerate, forming larger clusters, in a process known as catalyst sintering.^3^ As a result, the average particle size of the catalyst increases, leading to a decrease in the total metal surface area and the number of active sites available for catalysis.^4^ The development of catalytic systems where the active sites are dispersed throughout the surface of a support and possess a high resistance to sintering is one of the major challenges in modern industrial catalysis. For instance, in a typical cyclic reforming reactor running on a supported platinum catalyst, the catalyst is designed for regeneration after only a few days and needs to be entirely replaced after 4 to 5 years.^1^ Therefore, maintaining the catalyst’s activity and extending the time between catalyst replacements is a key challenge and of great industrial importance. In this context, polyoxometalates (POMs) offer a wide variety of solutions ranging from catalysts, nanoparticles, and active site stabilizers to nanosized entities serving as precursors for highly dispersed catalysts. For instance, polyoxopalladates (POPs)^35,36^ can serve as nanosized catalyst precursors where the POPs are immobilized on the surface of the support and reduced to form fairly monodisperse palladium metal nanoparticles, allowing the tuning of the active site dispersibility to a molecular level.^37^

The chemistry of the discrete wheel-shaped 48-tungsto-8-phosphate [P_8_W_48_O_184_]^40–^ (P_8_W_48_) has witnessed significant development in the last two decades. Contant and Tézé first reported the mixed potassium lithium salt of this polyanion in 1985.^5^ In 2005, Kortz’s group demonstrated that *d*-block metal ions can be incorporated into the central cavity by reporting the 20-copper­(II) derivative [Cu_20_Cl­(OH)_24_(H_2_O)_12_(P_8_W_48_O_184_)]^25–^ (Cu_20_P_8_W_48_).^6^ This fascinating polyanion paved the ground toward the development of an entire subclass of P_8_W_48_ with different types and numbers of metal ion guests in the central cavity. In 2007, Mialane reported the icosanuclear Cu^II^-azido derivative [Cu_20_(N_3_)_6_(OH)_18_P_8_W_48_O_184_]^24–^,^7^ and Pope reported the lanthanide-containing [Ln_2_(H_2_O)_10_(H_4_W_4_O_12_)_2_P_8_W_48_O_184_]^13–^,^8^ and together with Müller he reported a mixed-valent dodecavanadium-containing derivative.^9^ In 2008, the 16-Fe^III^-containing derivative [Fe_16_(OH)_28_(H_2_O)_4_P_8_W_48_O_184_]^20–^ was reported by Kortz and Müller,^10^ and two cobalt salts of P_8_W_48_ by Cronin.^11^ The bromo and iodo-centered derivatives of Cu_20_-Cl were reported in 2009.^12^ In the same year, Müller reported a 12-Mo-containing P_8_W_48_ encapsulating unprecedented neutral [Mo^V^_4_O_10_(H_2_O)_3_] aggregates.^13^ In 2010, Kortz reported the M_4_-containing family of P_8_W_48_ (M = Co^II^, V^V^, Mn^II^, and Ni^II^),^14^ Wang reported Co- and Ni-linked frameworks of M_1.5_P_8_W_49_ (M = Co^II^, Ni^II^),^15^ and Cronin reported a P_8_W_48_-based framework connected via manganese­(II) ions.^16,17^ In 2011, the latter group isolated two potassium-free salts of P_8_W_48_.^18^ Proust’s group reported two manganese­(II) derivatives, ([Mn_8_(H_2_O)_26_(P_8_W_48_O_184_)]^24–^ and [Mn_6_(H_2_O)_22_[WO_2_(H_2_O)_2_]_1.5_(P_8_W_48_O_184_)]^25–^),^19^ and Fang and Kögerler reported a very large assembly involving one P_8_W_48_ entity, all in the same year.^20^ In 2012, Kortz’s group reported the open-wheel [Fe_16_O_2_(OH)_23_(H_2_O)_9_Ln_4_(H_2_O)_20_(P_8_W_49_O_189_)]^11–^,^21^ and Floquet and Cadot reported the [K_4_[Mo_4_O_4_S_4_(H_2_O)_3_(OH)_2_]_2_(WO_2_)­(P_8_W_48_O_184_)]^30–^.^22^ In 2013 Yang et al. reported tetra-lanthanide and octa-manganese P_8_W_48_ derivatives,^23^ in 2014 Wang and Su reported some cobalt­(II)-linked P_8_W_48_ network assemblies,^24^ and in 2015 Kögerler’s group reported the Sn^II^-containing [(ClSn^II^)_8_P_8_W_48_O_184_]^17.5–^.^25^ In 2017 Kögerler’s group reported some organoarsonate derivatives,^26^ and in 2019 Kai-Yao Wang’s group reported the selenium­(IV) derivative [(SeO)_4_P_8_W_48_O_184_]^32–^.^27^ In the same year, Duval’s group reported a uranyl-containing P_8_W_48_ wheel, [(UO_2_)_7.2_(HCOO)_7.8_(P_8_W_48_O_184_)­Cl_8_]^41.4–^,^38^ and in 2020 Kortz reported the first peroxo-uranyl-containing P_8_W_48_ wheel and a detailed infrared and Raman spectroscopy study.^39^ This structure is related to the peroxo-uranyl-containing P_6_W_36_ ion reported in 2008 by the same group in which the P_8_W_48_ wheel is lacking a P_2_W_12_ unit, resulting in a U-shaped structure.^48^ In 2021, Yamaguchi and Suzuki reported two discrete manganese-containing P_8_W_48_ derivatives, the Mn_18_ and Mn_20_ derivatives, and studied their activity in oxidation catalysis.^40^ In 2022, Sokolov reported a platinum-containing P_8_W_48_ synthesized under hydrothermal conditions,^41^ Yamaguchi and Suzuki reported several copper-containing P_8_W_48_ compounds,^42^ and Du, Zang, and Yang reported several antimony- and arsenic-containing P_8_W_48_ wheels.^43^ In 2023, Yamaguchi and Suzuki reported the first silver-containing P_8_W_48_ in organic solvent, containing a record number of metals inside the cavity of the wheel to date.^44^ In 2024, Yang reported a novel multicomponent cluster comprising cationic Fe^III^ and Ce^III^ heterometals that was synthesized in the cavity of the P_8_W_48_.^46^ In 2025, Suzuki published a Pd-containing P_8_W_48_ which is used as a precursor for an encapsulated Pd cluster inside the cavity of P_8_W_48_ and explored its catalytic activity in highly selective hydrogenation reactions.^47^ A recent comprehensive review summarizing all POMs based on the P_8_W_48_ unit or its one-half (P_4_W_24_) or one-quarter (P_2_W_12_) units was published by Mal and Kortz.^45^ The only organo-noble-metal-containing P_8_W_48_ derivative, [(K­(H_2_O)}_3_{Ru­(*p*-cymene)­(H_2_O))_4_P_8_W_49_O_186_(H_2_O)_2_]^27–^, was reported in 2007.^28^

Here we report on the incorporation of IrCp* and RhCp* in the P_8_W_48_ wheel and the catalytic properties of these compounds.

## Experimental Section

K_28_Li_5_[H_7_P_8_W_48_O_184_]·92H_2_O was synthesized according to the reported procedure by Contant and Tézé.^5^ The purity was confirmed by FT-IR and ^31^P NMR spectroscopy. The (Ir^III^Cp*Cl_2_)_2_ and (Rh^III^Cp*Cl_2_)_2_ were synthesized according to the literature^29^ and the purity was confirmed by FT-IR and ^1^H as well as ^13^C NMR spectroscopy.

### Synthesis of Li_18_K_14_[{Rh­(Cp*)­(H_2_O)}_4_P_8_W_48_O_184_]·104H_2_O (**LiK-1**)

(Rh^III^Cp*Cl_2_)_2_ (C_20_H_30_Cl_4_Rh_2_, 0.009 g, 0.0140 mmol) and K_28_Li_5_[H_7_P_8_W_48_O_184_]·92H_2_O (0.100 g, 0.0068 mmol) were dissolved in a mixture of 3 mL of 1 M lithium acetate solution (pH 6.0) and 250 μL of a 1 M lithium perchlorate solution. This solution was heated in a water bath at 75–80 °C for 30 min. The dark-orange solution was allowed to cool to room temperature and left for crystallization in an open vial. Red-orange crystals of **LiK-1** formed after 2–3 days, which were collected by filtration and air-dried (yield: 65 mg, 62%). FTIR (1% KBr pellet): ν = 2924 (w), 1629 (s), 1383 (w), 1136 (s), 1081 (s), 1016 (m), 927 (s), 808 (s), 686 (s), 573 (w), 532 (w), 458 (w). Elemental analysis, calculated (found): K 3.53 (3.43), Li 0.81 (0.78), Rh 2.65 (2.58), P 1.60 (1.65), W 56.88 (56.32).

### Synthesis of Li_16_K_16_[{Ir­(Cp*)­(H_2_O)}_4_P_8_W_48_O_184_]·101H_2_O (**LiK-2**)

(Ir^III^Cp*Cl_2_)_2_ (C_20_H_30_Cl_4_Ir_2_, 0.011 g, 0.014 mmol) and K_28_Li_5_[H_7_P_8_W_48_O_184_]·92H_2_O (0.100 g, 0.0068 mmol) were dissolved in a mixture of 3 mL of 1 M lithium acetate solution (pH 4.0) and 250 μL of a 1 M lithium perchlorate solution. This solution was heated in a water bath at 75–80 °C for 30 min. The light-orange solution was allowed to cool to room temperature and left for crystallization in an open vial. Orange-yellow needle-shaped crystals of **LiK-2** formed after 2–3 h, which were collected by filtration after 3 days and air-dried (yield: 58 mg, 55%). FTIR (1% KBr pellet): ν = 2924 (w), 1626 (s), 1384 (w), 1136 (s), 1086 (s), 1020 (m), 928 (s), 808 (s), 688 (s), 573 (w), 532 (w), 463 (w). Elemental analysis, calculated (found): K 3.92 (3.90), Li 0.44 (0.45), Ir 4.83 (4.82), P 1.56 (1.61), W 55.43 (55.82).

### X-ray Diffraction

Single crystals of the two compounds were mounted in a Hampton cryoloop in light oil for data collection at a low temperature (100 K). The X-ray data were collected on a Bruker X8 APEX II CCD diffractometer with kappa geometry and Mo Kα radiation (λ = 0.71073 Å). Data integration and routine processing were performed by using the SAINT software suite. Further data processing, including absorption corrections from equivalent reflections, was performed using SADABS.^19^ Direct methods (SHELXS97) solutions successfully located the W atoms, and successive Fourier syntheses (SHELXL97) revealed the remaining atoms.^20^ Refinements were full-matrix least-squares against *F* ^2^ using all of the data. The counter cations and waters of hydration were modeled with varying degrees of occupancy, which is common in polyoxotungstate structures. Crystallographic data for both compounds are summarized in Table 1. Further details on the crystal structure investigations may be obtained free of charge under CCDC 2518808 (**LiK-1**) and 2518809 (**LiKNa-2**) from The Cambridge Crystallographic Data Centre via http://www.ccdc.cam.ac.uk/data_request/cif.

**Table 1:** Single Crystal XRD Data and Structure Refinement Parameters for **LiK-1** and **LiK-2**

Empirical formula	K_14_Li_18_Rh_4_C_40_H_276_P_8_W_48_O_292_	K_16_Li_16_Ir_4_C_40_H_270_P_8_W_48_O_289_ ^a^
Formula weight, g/mol	7046.24^b^	29345^b^
Crystal system	Triclinic	Triclinic
Space group	*P*-1	*P*-1
*a*, Å	19.903(2)	23.3584(19)
*b*, Å	21.288(2)	28.210(2)
*c*, Å	22.597(3)	29.917(2)
α, °	69.161(3)	68.540(2)
β, °	83.553(4)	89.697(3)
γ, °	78.353(3)	69.867(2)
Volume, Å^3^	8755.3(17)	17061(2)
*Z*	2	1
*D*_calc_, g/cm^3^	2.673	2.856
Absorption coefficient, mm^–1^	16.155	18.227
*F*(000)	6178	12793
Theta range for data collection, °	1.340 to 25.000	1.540 to 27.609
Completeness to Θ_max_	100%	99.9%
Index ranges	–23 ≤ *h* ≤ 23, −25 ≤ *k* ≤ 25, −26 ≤ *l* ≤ 26	–30 ≤ *h* ≤ 30, −36 ≤ *k* ≤ 36, −38 ≤ *l* ≤ 38
Reflections collected	187762	324805
Independent reflections	30813	78606
*R*(int)	0.1024	0.1749
Absorption correction	Semiempirical from equivalents	Semiempirical from equivalents
Data/restraints/parameters	30813/822/1504	78606/1608/3032
Goodness-of-fit on F^2^	1.018	1.004
*R*_1_ ^c^ w*R*_2_ ^d^ (*I* > 2σ(*I*))	*R*_1_ = 0.0765, w*R*_2_ = 0.2221	*R*_1_ = 0.0758, w*R*_2_ = 0.1940
*R*_1_ ^c^ w*R*_2_ ^d^ (all data)	*R*_1_ = 0.1077, w*R*_2_ = 0.2467	*R*_1_ = 0.1536, w*R*_2_ = 0.2372

a We located some sodium ions by XRD in this structure, although we never added a sodium-containing reagent. The sodium ions must have originated from the ultrapure water used as solvent.

b The formula and molar mass are based on bulk elemental analysis.

c *R*_1_ = ∑∥*F*_0_| – |*F*_*c*_∥/∑|*F*_0_|.

d w*R*_2_ = [∑*w*(*F*_0_^2^ – *F*_*c*_^2^)^2^/∑*w*(*F*_0_^2^)^2^ ]^1/^ ^2^.

### NMR Spectroscopy

The solution NMR measurements were performed on a 400 MHz JEOL ECS instrument using 5 mm tubes in a probe tuned to the corresponding frequency (162 MHz for ^31^P and 100.6 MHz for ^13^C). The isolated salts of the polyanions **1** and **2** (ca. 25 mg) were dissolved in 0.5 mL D_2_O and the spectra were obtained after a few hours of measurement time at room temperature.

### Synthesis of SBA15

The synthetic procedure of SBA15 was adapted from the originally published procedures.^30,31^ In a typical synthesis, 120 g of P_123_ (*M*_n_ ∼ 5,800, Sigma-Aldrich) were stirred in a mixture of 3.6 L of water and 100 mL of 37% HCl_aq_ until complete dissolution (∼4 h). To this solution, 270 mL of TEOS were added dropwise. The resulting solution was stirred in a water bath for 16 h at 36 °C, and then aged at 95 °C under static conditions for 72 h. The white precipitate was collected by filtration, dried in air for 2 days, followed by calcination at 550 °C for 6 h with a heating rate of 1 °C·min^–1^ to remove the template.

### Synthesis of Aminopropyl-Modified SBA15

SBA15 (33 g) and (3-aminopropyl)­triethoxysilane (apts) (18 mL) were refluxed for 5 h in 1 L of toluene and filtered at room temperature. The resulting white powder was dried at 100 °C for 5 h.

### Catalyst Preparation

Twenty wt % **1**@SBA15 (containing 0.5 wt % Rh) and **2**@SBA15 (containing 1 wt % Ir) were prepared by stirring one weight equivalent of the POM with four weight equivalents of SBA15-apts in water for 16 h at room temperature. The mixture was filtered and washed with water. The absence of color in the filtrate indicates that the POM was quantitatively loaded onto the support. The supported catalyst was then dried in air for 3 days, followed by an air-calcination step at 400 °C for 4 h with a heating ramp of 0.5 °C·min^–1^.

### Nitrogen Adsorption

N_2_ adsorption (physisorption) measurements were performed at 77 K on a NOVA 4000e surface area and pore size analyzer from Quantachrome. The samples were placed in 9 mm Quantachrome cells and degassed under vacuum at 100 °C for at least 6 h prior to the measurements. The surface area was estimated using the Brunauer–Emmett–Teller (BET) theory, and the pore size was determined using the Barrett–Joyner–Halenda (BJH) method.^32,33^

### Hydrogenation Reactions

The hydrogenation reactions were carried out using a Microactivity-EFFI unit purchased from PID Eng&Tech (Spain) equipped with a fixed-bed stainless steel reactor with a 9.1 mm internal diameter and coupled with an Agilent 6890 GC-FID equipped with a switching valve from VICI for online analysis. The identification of the reaction products was performed using a hydrocarbon standards kit (PIANO kit: paraffins, isoparaffins, aromatics, naphthenes, olefins) purchased from Sigma-Aldrich. The details of the method used on the GC for analysis are described in the Supporting Information.

In a typical reaction, 1 g of calcined catalyst was loaded into the reactor. A sufficient quantity of SiC was used as a diluent before and after the catalyst to ensure that the latter was in the isothermal zone of the reactor. Prior to the reactions, the catalyst was activated by heating to 425 °C for 1 h at a rate of 2 °C·min^–1^. The activation was performed under a hydrogen flow of 200 mLN·min^–1^ at 7 bar. During the activation, the noble metal oxides are reduced to form the active catalyst.

## Results and Discussion

Here we demonstrate a strategy to stabilize noble metal nanoparticles by grafting their respective precursors onto discrete polyoxometalates (POMs), in an attempt to reduce sintering and prolong the catalyst lifetime. The wheel-shaped 48-tungsto-8-phosphate [P_8_W_48_O_184_]^40–^ (P_8_W_48_) which has a ca. 1 nm wide cavity (and hence a very large lacunary site) is hydrolytically stable and robust in a wide pH range and thermally stable. These criteria render this superlacunary polyanion a suitable host to graft noble metal ions and hence become highly relevant for catalytic applications. If the noble metal ions after reduction remain encapsulated within the P_8_W_48_ ring, the agglomeration of noble metal nanoparticles could be limited or even inhibited, allowing to form a highly dispersed supported catalyst (e.g., for hydrogenation reactions). In other words, superlacunary P_8_W_48_ offers the unique opportunity of tuning the catalyst composition and structure on a molecular level, which allows for ultimate control over the composition and the dispersion of the catalyst. In addition, the anionic nature of P_8_W_48_, its aqueous solubility, and solution stability are important factors that facilitate the immobilization and incorporation of catalysts onto solid supports. As a discrete catalyst precursor, we decided to use the organometallic (MCp*Cl_2_)_2_ (M = Rh^III^, Ir^III^) and graft it onto the discrete, fully inorganic P_8_W_48_ polyanion host.

The novel polyanions [{M­(Cp*)­(H_2_O)}_4_P_8_W_48_O_184_]^32–^ (**1**, M = Rh; **2**, M = Ir) were prepared in aqueous acidic acetate solutions (pH 6 for **1** and pH 4 for **2**) under mild reaction conditions using a one-pot synthetic procedure. Single-crystal X-ray diffraction revealed that the structures **LiK-1** and **LiK-2** crystallize in the triclinic space group *P*-1 and that four MCp* (M = Rh, Ir) entities are connected to the cavity of the P_8_W_48_ host via two M–O­(W) bridges (see Figure 1). More precisely, the organometallic guests are located opposite to one another, leaving the other four vacant sites in the cavity of the polyanion free, resulting in a structure with idealized *D*_*2h*_ symmetry of **1** and **2**. Also, a terminal water molecule is connected to the metal center M, in addition to the Cp* ligand. The M–O­(W) bond lengths range from 2.100 to 2.149 Å for rhodium derivative **1** and 2.113 to 2.146 Å for iridium derivative **2**. The four bulky Cp* ligands are pointing away from the cavity of the P_8_W_48_ wheel, probably due to steric effects. As a result, only four MCp* units can bind to the cavity of the wheel-shaped host in which they sit in pairs on opposite sides. The M–OH_2_ bonds also point away from the polyanions and those on opposite faces of the wheel are essentially coaligned. It is important to mention that both polyanions **1** and **2** are slightly distorted compared to the P_8_W_48_ precursor. The two M–O­(W) bonds bridging two P_2_W_12_ units decrease the distance between them, which leads to an overall distortion of the structure. Such an allosteric phenomenon was previously observed for the organo-Ru and Se derivatives of P_8_W_48_.^27,28^

**Figure 1:**
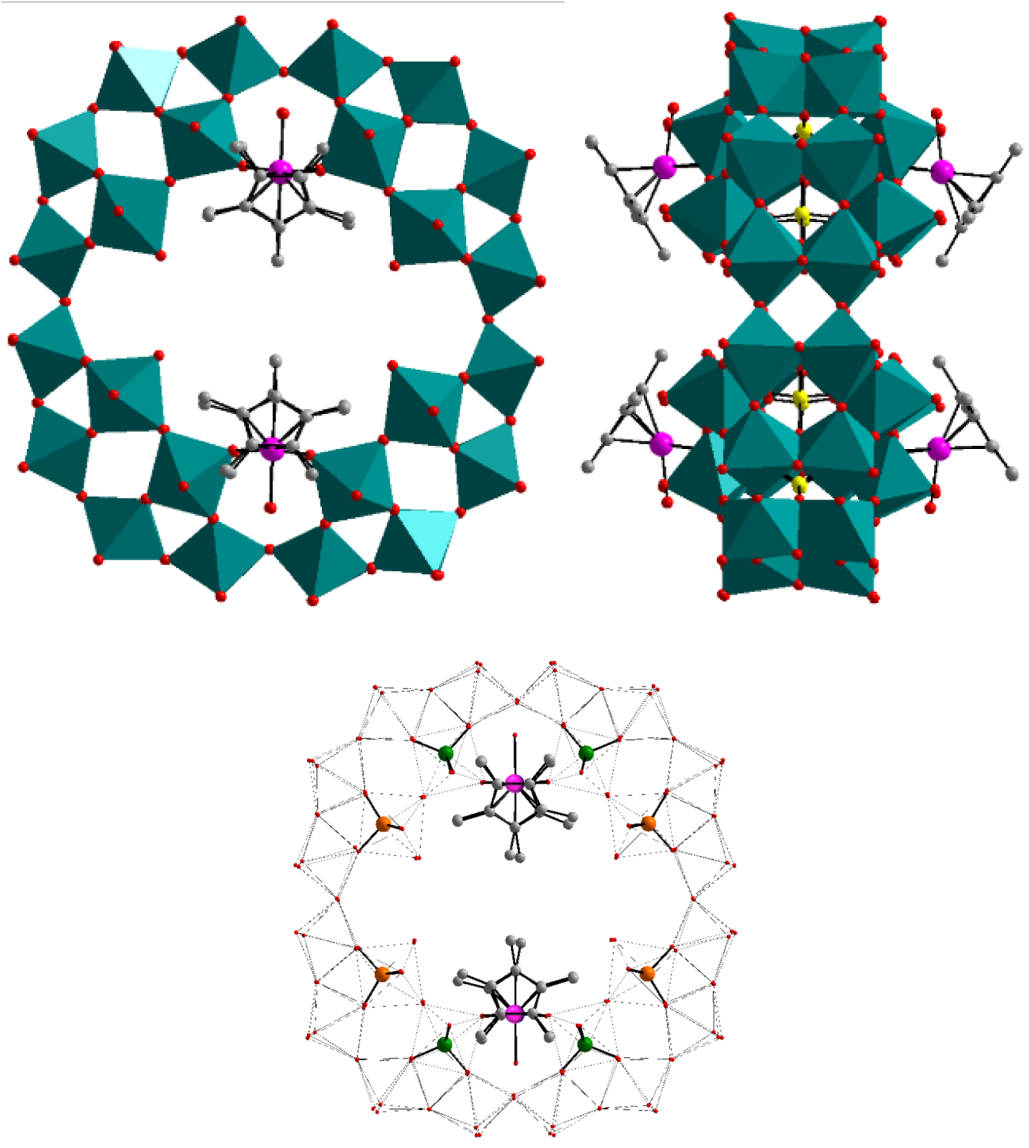
Combined polyhedral and ball-and-stick representations of polyanions **1** and **2**; top view (upper left) and side view (upper right). The ball-and-stick representation at the bottom highlights the two sets of magnetically inequivalent P atoms. Color code: Rh/Ir (pink), WO_6_ (green), P (yellow), O (red), and C (gray). Hydrogens are not shown.

The rhodium derivative **1** was prepared by reacting two equivalents of the dimeric organometallic precursor (RhCp*Cl_2_)_2_ with one equivalent of P_8_W_48_ at pH 6 in a lithium acetate solution. The iridium derivative was prepared following the same procedure but at pH 4. So the best yields of both polyanions **1** and **2** were obtained when two equivalents of the organometallic precursors were reacted with one equivalent of P_8_W_48_, although the stoichiometry in the products suggests otherwise. We also tried the stoichiometric ratio of 4:1, but this resulted in the formation of extended frameworks of **1** connected via RhCp* bridges. Such behavior was not observed for **2**, where the ratio of IrCp* to P_8_W_48_ did not lead to additional products. According to the XRD data for **LiK-2**, one extra tungsten atom is disordered over the four remaining vacant sites in the cavity of the polyanion, which is also reflected in the results of the elemental analysis, where the weight percent of W was found to be very slightly higher than the theoretical value calculated. Therefore, some of the **LiK-2** units are found to contain an extra tungsten in the cavity of the P_8_W_48_ disordered over the four empty positions. This was realized by analyzing the X-ray data of **LiK-2**. The compound containing extra tungsten in the cavity can be roughly formulated as [{Ir­(Cp*)­(H_2_O)}_4_{WO_2_(OH_2_)_2_}­P_8_W_48_O_184_]^30–^. In spite of significant efforts, we were unable to obtain analytically 100% clean material. The presence of the additional tungsten in the cavity was also reflected in the ^31^P NMR spectrum of **2** by an additional peak accounting for about 20% (*vide infra*). As no extra tungsten was added in the reaction, it most likely originates from *in situ* decomposition of a small amount of P_8_W_48_, as also observed previously for the organoruthenium-substituted P_8_W_48_ reported in 2007.^28^

The ^31^P NMR spectrum of **LiK-1** dissolved in water exhibits two closely spaced signals at −5.65 and −5.76 ppm, respectively (see Figure 2), one corresponding to the four equivalent P atoms situated in the proximity of the Rh centers and the other to the four more distant P atoms (see Figure 1). There is a very small (<1%) peak at −6.18 ppm, corresponding to an unknown impurity. On the other hand, the ^31^P NMR spectrum of **LiK-2** dissolved in water also exhibits two closely spaced signals at −4.91 and −5.06 ppm, respectively (see Figure 3), corresponding to polyanion **2**. The additional two overlapping peaks at about 4.0 ppm are most likely due to the derivative of **2** containing an extra tungsten atom in the central cavity of P_8_W_48_ (*vide supra*).

**Figure 2:**
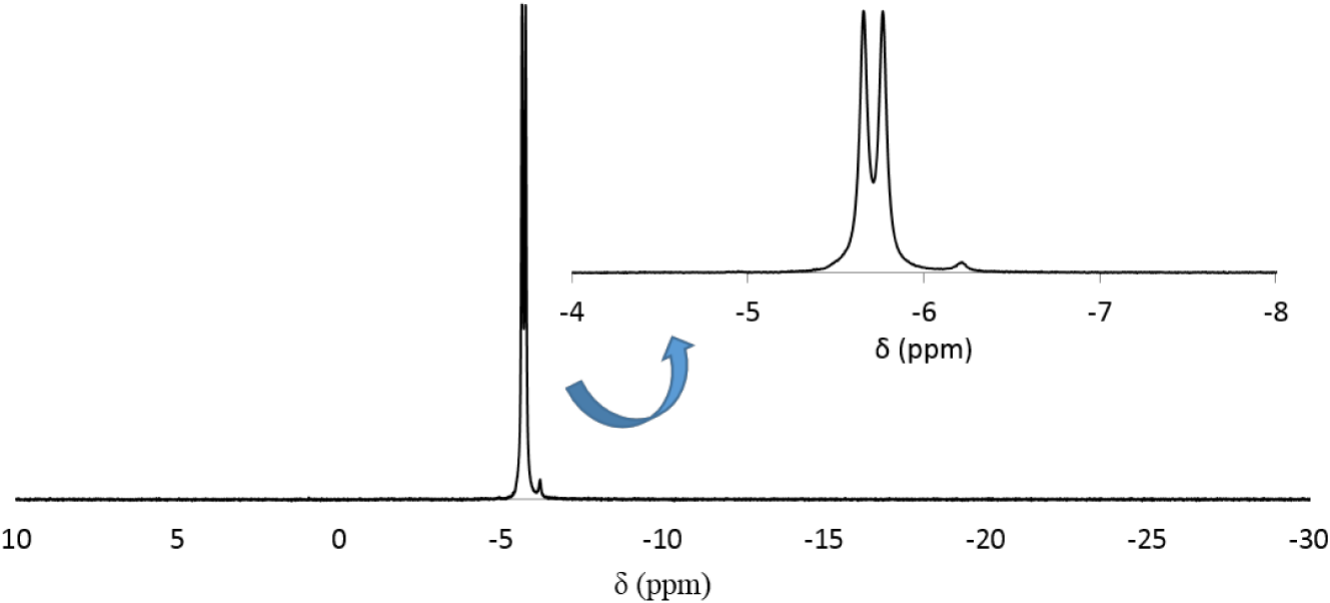
^31^P NMR spectrum of **LiK-1** dissolved in H_2_O.

**Figure 3:**
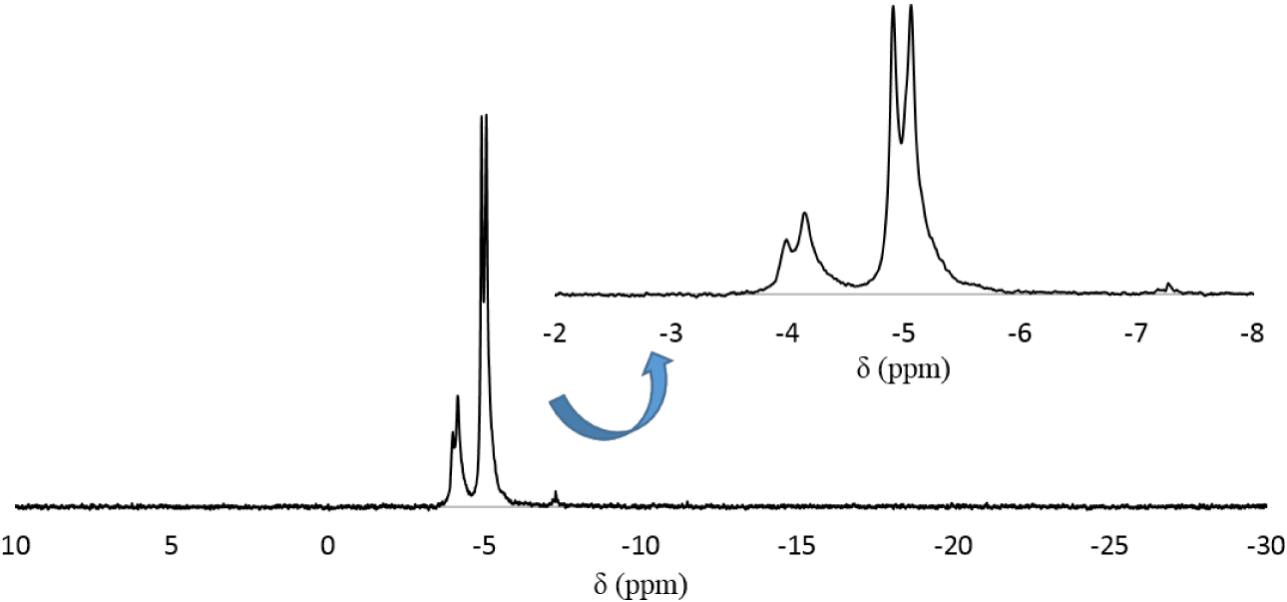
^31^P NMR spectrum of **LiK-2** dissolved in H_2_O.

The ^13^C NMR spectrum of **LiK-1** showed two signals at 8.8 and 94.1 ppm, respectively (Figure 4), with the former being due to the five methyl groups of the Cp* ligand and the latter due to the five carbon atoms of the Cp* cycle. It is important to mention that the Rh–C coupling of the (RhCp*Cl_2_)_2_ unit is not clearly resolved, unlike the organometallic precursor itself in dichloromethane (DCM) showing a sharp doublet. Interestingly, when the ^13^C NMR solution of **LiK-1** was measured at ca. 0 °C, the broad singlet observed at room temperature splits and results in the expected doublet clearly showing the Rh–C coupling (Figure S3).

**Figure 4:**
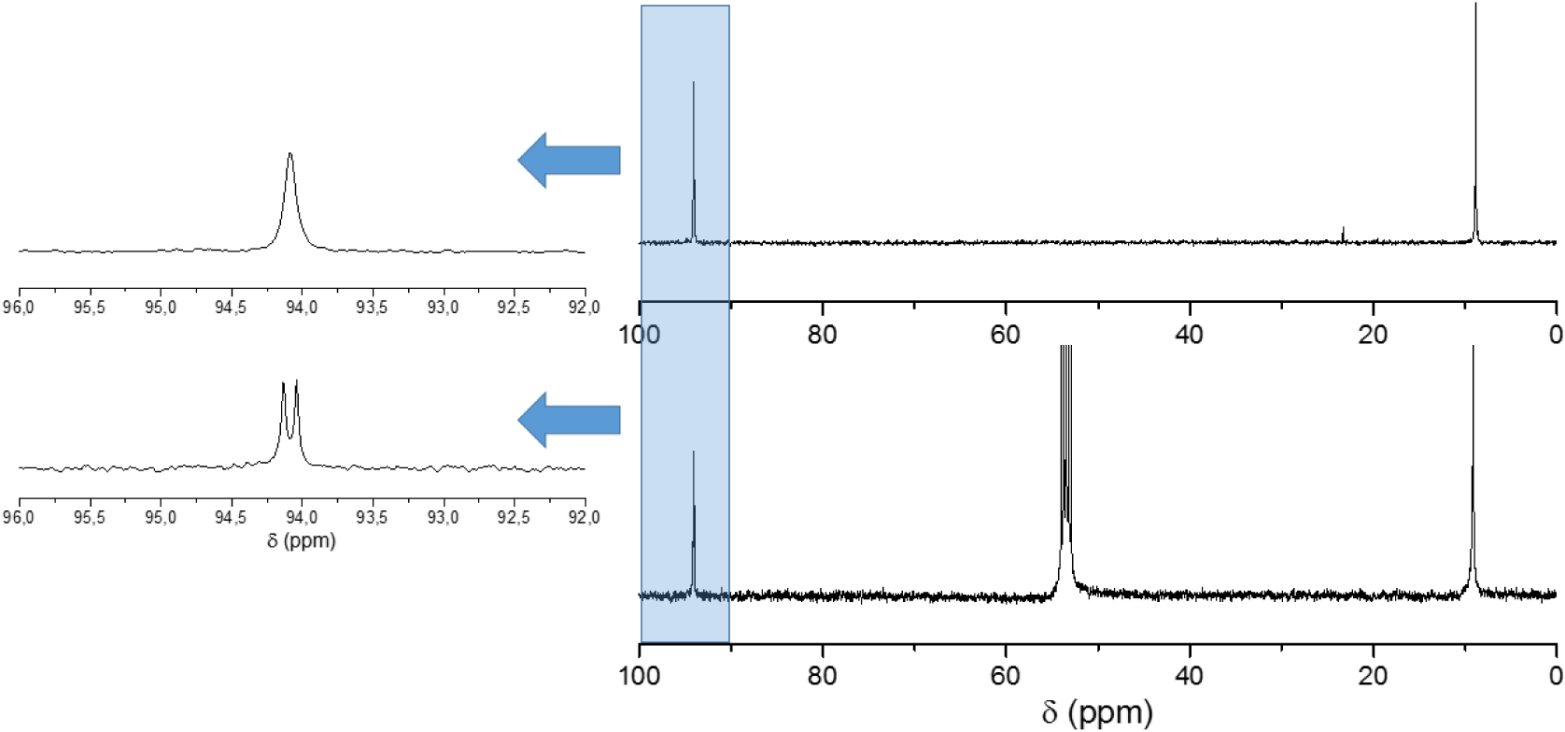
^13^C NMR spectra of **LiK-1** in H_2_O (top) and neat (RhCp*Cl_2_)_2_ in dichloromethane (DCM) (bottom).

The ^13^C NMR spectrum of **LiK-2** showed the expected two signals at 9.5 and 84.4 ppm, respectively, whereas the (IrCp*Cl_2_)_2_ precursor in DCM exhibited signals at 9.1 and 86.2 ppm, respectively (Figure 5).

**Figure 5:**
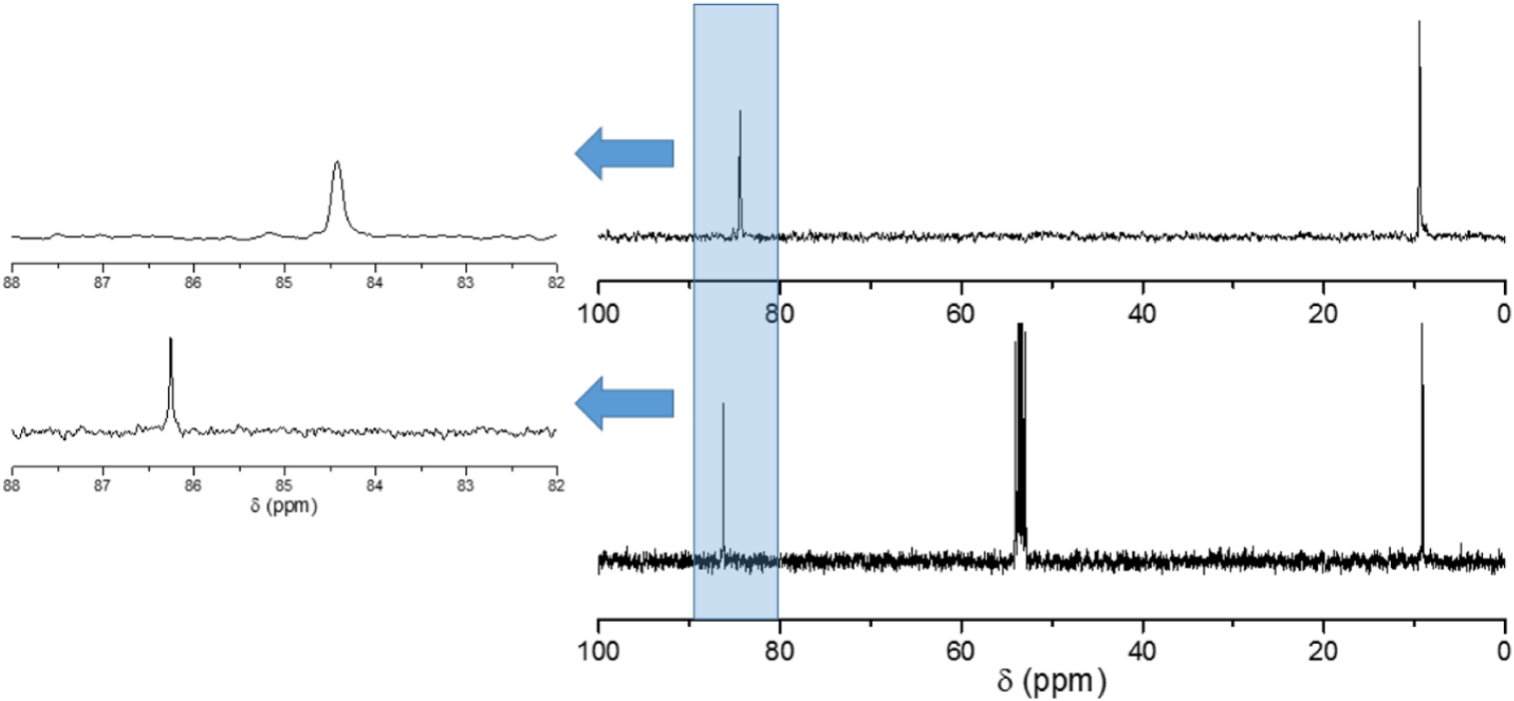
^13^C NMR spectra of **LiK-2** in H_2_O (top) and (IrCp*Cl)_2_ in DCM (bottom).

The fingerprint regions of the FT-IR spectra (see Figures S1 and S2) of both polyanion salts **LiK-1** and **LiK-2** are almost identical to that of the P_8_W_48_ precursor salt, which is common for this large compound. The vibrations of the dense W–O network of the P_8_W_48_ host most likely mask the Ir–O and Rh–O vibrations. The C–H stretching vibrations of the Cp* groups were clearly observed between 2800 and 3000 cm^–1^.

Thermogravimetric analysis (TGA) on **LiK-1** and **LiK-2** showed that both compounds are thermally stable up to 350 °C (Figures S4 and S5). Beyond this temperature, the Cp* group detaches from the structure. The weight loss between 350 and 600 °C corresponds to the weight of four Cp* units, which is consistent with the elemental analysis data. The number of crystal waters reported in the formula units was also calculated from the TGA data (and elemental analysis) using the weight loss between room temperature and 200 °C. Additionally, Figure S6 shows a comparison of the thermal stability of the (IrCp*Cl_2_)_2_ dimer and that of **LiK-2**. It is evident that coordination of the IrCp* group to the P_8_W_48_ wheel results in increased thermal stability. The loss of Cp* starts at 280 °C for the neat organometallic dimer but increases to ca. 400 °C for **LiK-2**.

In order to investigate the catalytic activity of **1** and **2**, we decided to immobilize the compounds on the mesoporous support SBA15-apts, resulting in **1**@SBA15 and **2**@SBA15, respectively, followed by air calcination for generating Rh/Ir-oxide nanoparticles, which upon activation under hydrogen flow would result in Rh/Ir nanoparticles. The calcination step results in the loss of the Cp* groups from the polyanion, as well as the aminopropyl group of the SBA15-apts support. After immobilization and calcination, the supported catalysts were then characterized by N_2_-adsorption.

The N_2_-adsorption data show a decrease in both the surface area and the pore volume of the support after modification with apts (Figure 6). A further decrease in the surface area and pore volume was observed upon immobilization of the POMs on the modified SBA15. After calcination, these values increase again due to the loss of the aminopropyl “arms” from the surface of the silica which was also confirmed by TGA measurements. On the other hand, the thermal analysis of the neat (MCp*Cl_2_)_2_ dimers vs **LiK-1** and **LiKNa-2** showed that grafting MCp* in the cavity of the P_8_W_48_ host increases the thermal stability of the organometallic units (*vide supra*). Nevertheless, under calcination conditions (400 °C for 4 h), TGA demonstrated that the Cp* groups are removed.

**Figure 6:**
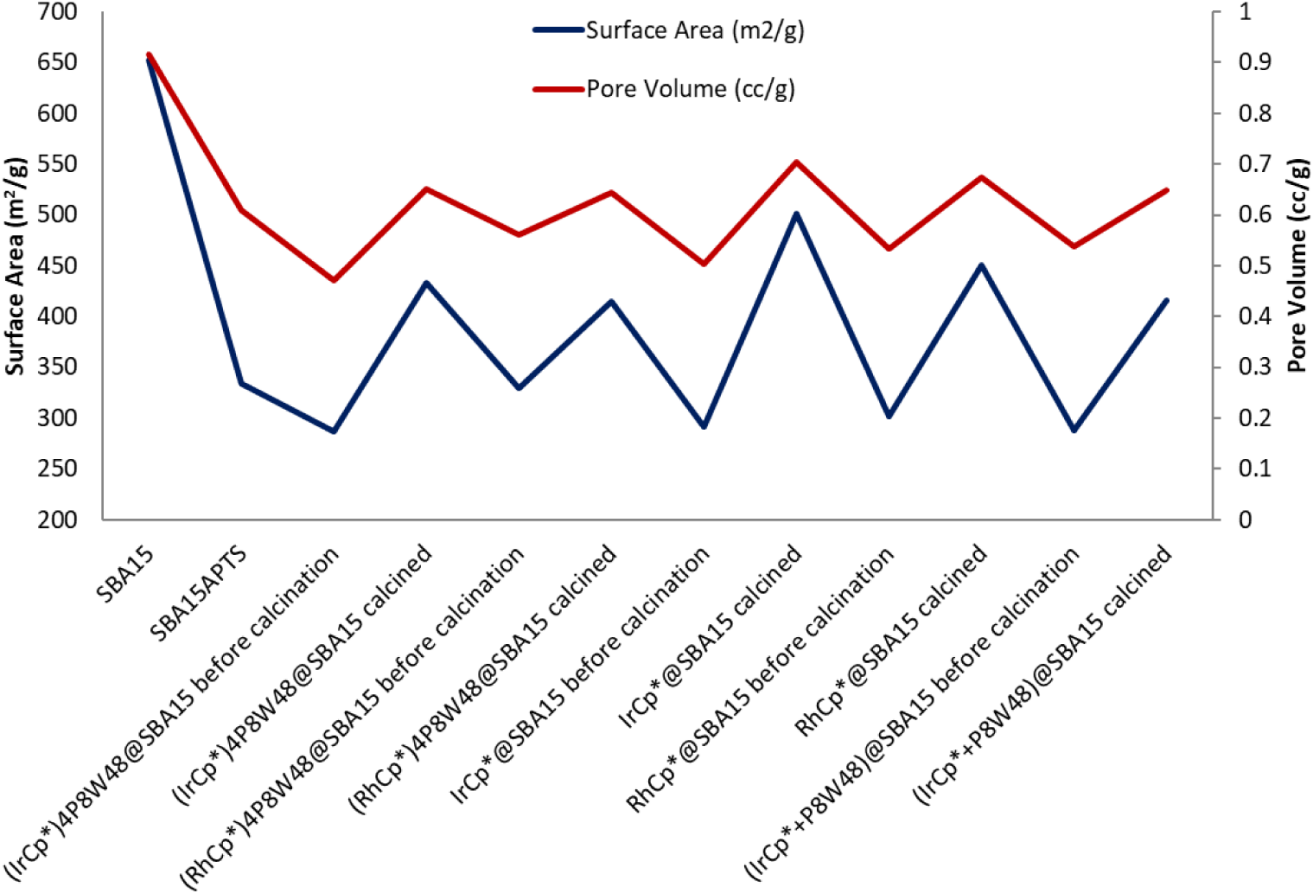
Surface areas of various supported catalysts discussed in this work.

The catalytic activity of organo-Rh- and Ir-substituted polyanions **1** and **2** was initially investigated for the hydrogenation of aromatics. The 0.5 wt % Rh- and 1 wt % Ir-loaded catalysts (both catalysts contained the same molar equivalents of noble metal) showed high activity in the hydrogenation of olefins. First, *o*-xylene was tested as a model substrate. The conversion of *o*-xylene and the selectivity to the reduced c*is* vs *trans* dimethylcyclohexane isomers are shown in Figures 7 and 8, respectively, for the supported **1**@SBA15 and **2**@SBA15 precatalysts. Both materials showed high activity between 150 and 250 °C. At lower reaction temperatures, the selectivity toward the *cis*-dimethylcyclohexane is higher than for the *trans* isomer. For the Rh-based catalyst, the *cis/trans* ratio decreased from 2.5 at 150 °C to about 0.3 at 250 °C and remained as such until 330 °C. On the other hand, for the Ir-analogue, the *cis/trans* ratio decreased from 12 at 150 °C to 1 at 250 °C and then decreased further from 1 to 0.5 between 250 and 330 °C. These findings are fully consistent with the *cis*–*trans* isomerism theory. At higher temperatures, the kinetic product (*cis*) is less favored compared to the thermodynamic (*trans*) product. In addition, upon increasing the reaction temperature beyond 250 °C, the decrease in the selectivity toward dimethylcyclohexane is due to cracking as well as ring opening (RO) of the hydrogenation products.

**Figure 7:**
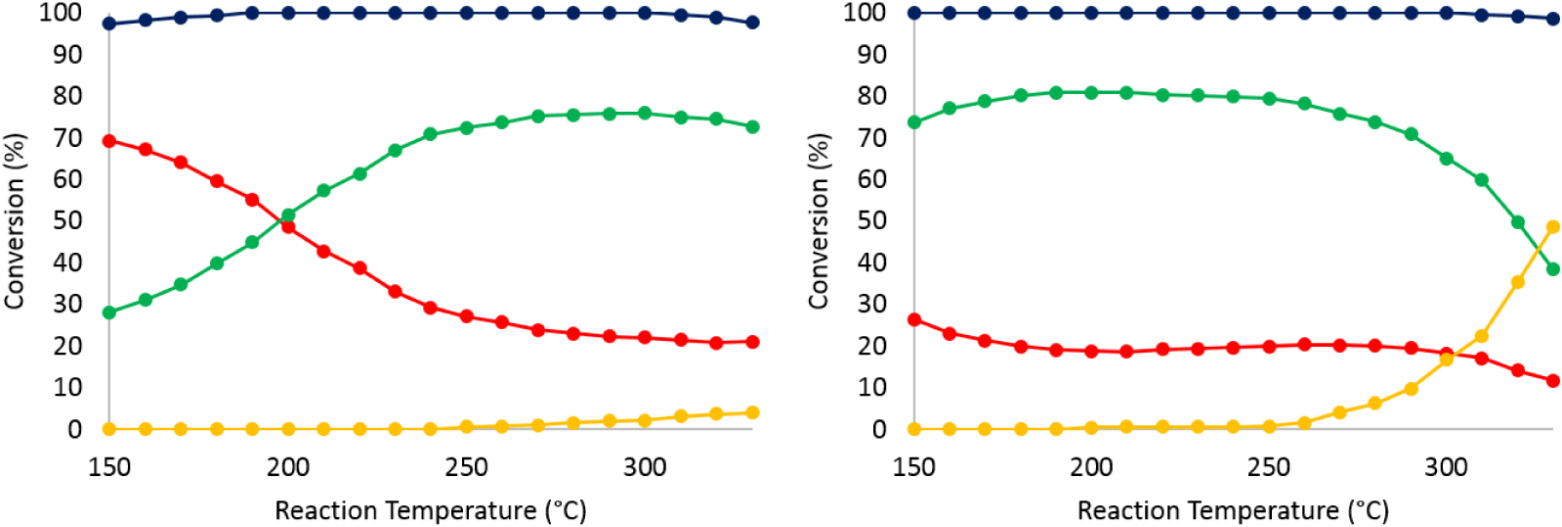
Conversion of *o*-xylene on **1**@SBA15 (left) and the reference RhCp*@SBA15 (right). Total conversion (blue), conversion to *cis-*dimethylcyclohexane (red), conversion to *trans*-dimethylcyclohexane (green), and conversion to ring-opening and cracking products (yellow). Reaction conditions: Feed: 0.5 M *o-*xylene in hexane, feed flow = 0.05 mL/min; H_2_ flow = 22.5 mLN/min; pressure = 28 bar.

**Figure 8:**
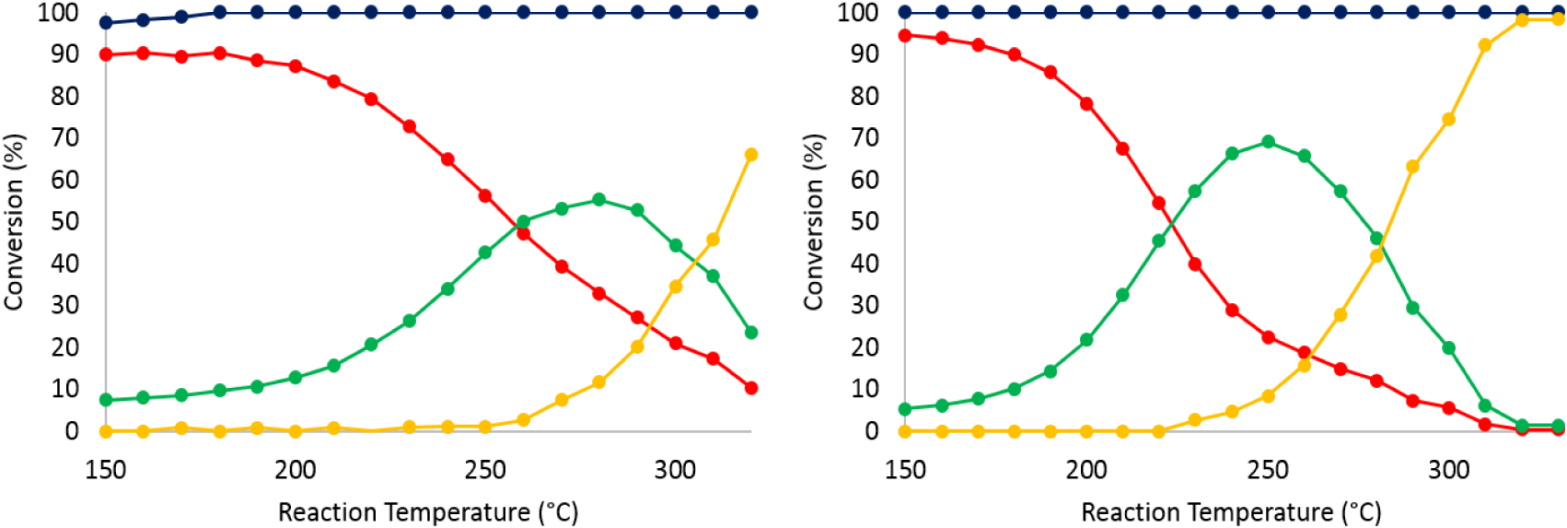
Conversion of *o*-xylene on **2**@SBA15 (left) and the reference IrCp*@SBA15 (right). Total conversion (blue), conversion to *cis-*dimethylcyclohexane (red), conversion to *trans*-dimethylcyclohexane (green), and conversion to ring-opening and cracking products (yellow). Reaction conditions: Feed: 0.5 M *o-*xylene in hexane, feed flow = 0.05 mL/min; H_2_ flow = 22.5 mLN/min; pressure = 28 bar.

It can be observed that the iridium-based precatalyst **2**@SBA15 showed much higher selectivity to RO products than the Rh-analogue **1**@SBA15, which is consistent with the literature.^34^ In order to compare the effect of RhCp* and IrCp* being grafted inside the cavity of P_8_W_48_ supported on SBA15-apts vs neat RhCp* and IrCp* supported on SBA15-apts, the latter two materials were prepared via the wet impregnation technique and calcined under the same reaction conditions. Figure 7 shows that both **1**@SBA15 and RhCp*@SBA15 exhibited high catalytic activity. However, the difference in the selectivity requires more attention. The *cis/trans* ratio for **1**@SBA15 varied as mentioned earlier, while in the case of RhCp*@SBA15, this ratio remained almost unchanged. Additionally, the conversion to ring-opening and dealkylation products is significantly higher for the latter (50% for RhCp*@SBA15 vs only 4% for **1**@SBA15 at 330 °C). The same observation can be made by comparing the data obtained from the reactions over **2**@SBA15 and IrCp*@SBA15 (Figure 8). However, the selectivity to ring-opening and cracking products in IrCp*@SBA15 is only twice as high as that of **2**@SBA15 at a given temperature, whereas for the Rh-analogues, the difference is an order of magnitude higher.

The advantageous effect of grafting MCp* (M = Rh, Ir) in the cavity of the P_8_W_48_ wheel is further demonstrated by independently and successively immobilizing P_8_W_48_ and IrCp* on SBA15 and comparing its performance to that of **2**@SBA15 and IrCp*@SBA15. The (IrCp* + P_8_W_48_)@SBA15 where the organoiridium complex and the P_8_W_48_ polyanion are independently and randomly distributed on the surface of the SBA15 exhibited an almost identical behavior as the IrCp*@SBA15 (Figure 9). This implies that the structural and compositional features of the discrete organometallic-P_8_W_48_ derivatives **1** and **2** are superior to those of a mixture of the individual components (organometallic precursor and P_8_W_48_) from a catalytic point of view. It is also worth mentioning that the same catalytic reactions were performed by loading the SBA15 support alone as well as P_8_W_48_@SBA15 to establish a baseline. In both cases, there was no catalytic activity observed under the same reaction conditions, indicating the inactivity of the support alone and supported P_8_W_48_ in these reactions.

**Figure 9:**
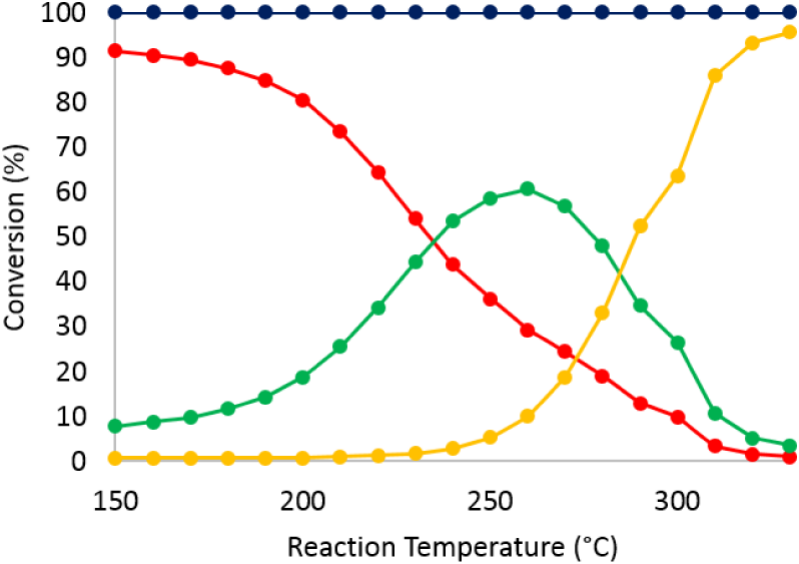
Conversion of *o*-xylene on (IrCp* + P_8_W_48_)@SBA15. Total conversion (blue), conversion to *cis-*dimethylcyclohexane (red), conversion to *trans*-dimethylcyclohexane (green), and conversion to ring-opening and cracking products (yellow). Reaction conditions: Feed: 0.5 M *o-*xylene in hexane, feed flow = 0.05 mL/min; H_2_ flow = 22.5 mLN/min; pressure = 28 bar.

Furthermore, the influence of increasing the feed flow rate on the conversion of *o-*xylene and selectivity to the *cis*- or *trans*- products was investigated for both **1**@SBA15 and **2**@SBA15. As expected, the conversion of *o-*xylene is lowered when increasing the feed flow, as shown in Figure 10. In addition, the impact of increasing the flow of H_2_ on the reaction was also assessed. Multiplying the H_2_ flow by 1.5, 2, 3, or 4 did not have any significant impact on the outcome of the reaction.

**Figure 10:**
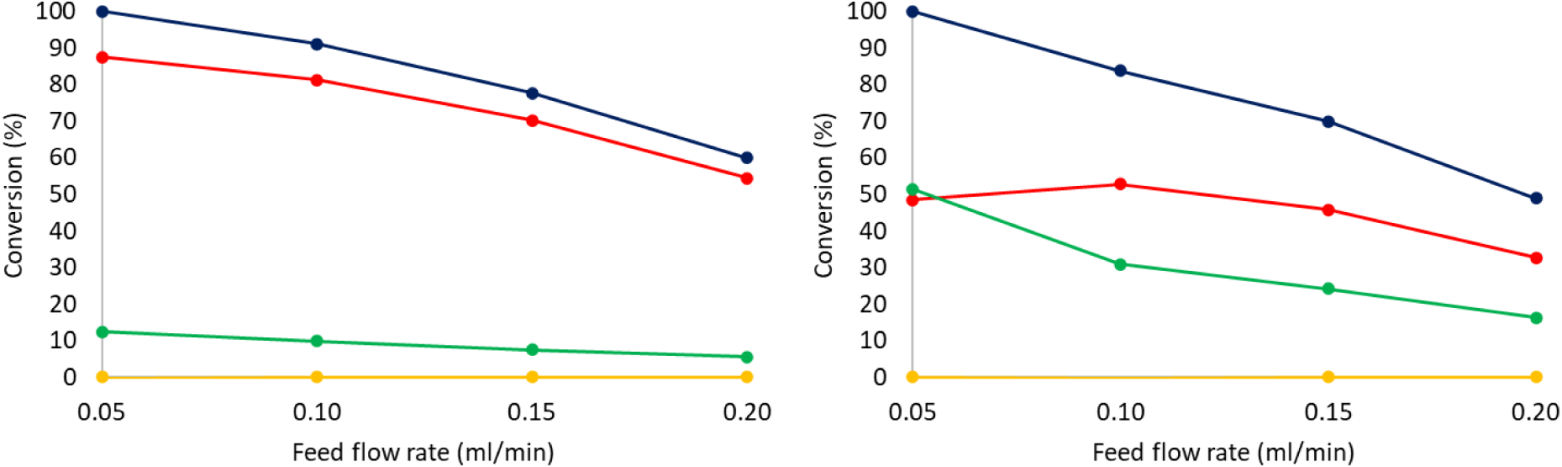
Conversion of *o*-xylene on **2**@SBA15 (left) and **1**@SBA15 (right) vs feed flow rate. Total conversion (blue), conversion to *cis-*dimethylcyclohexane (red), conversion to *trans*-dimethylcyclohexane (green), and conversion to ring-opening and cracking products (yellow). Reaction conditions: Feed: 0.5 M *o-*xylene in hexane, reaction temperature: 200 °C; H_2_ flow = 25 mLN/min; pressure = 28 bar.

The on-stream stability of **1**@SBA15 was evaluated for about 150 h during which the feed flow rate was doubled after about 110 h. During the 150 h on stream, the catalyst exhibited negligible loss of activity which demonstrates its stability (Figure 11).

**Figure 11:**
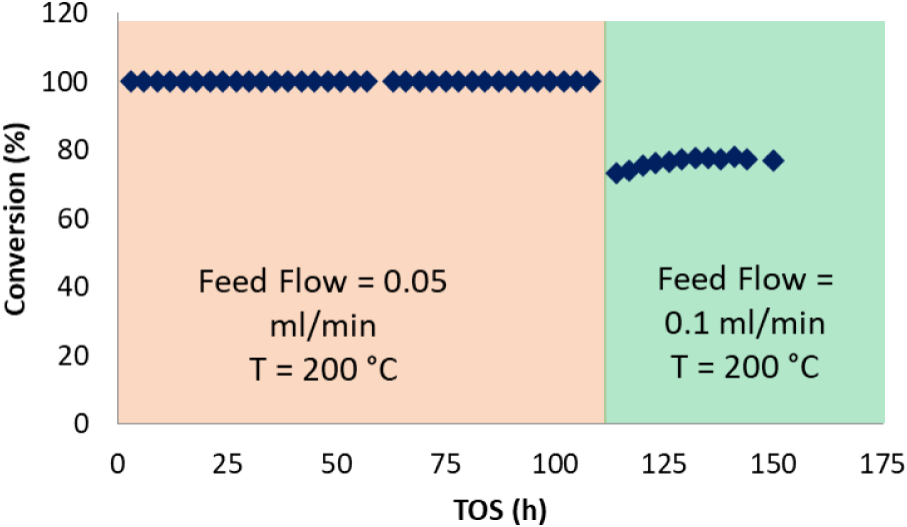
Conversion of *o*-xylene on **1**@SBA15 vs time on stream (TOS). Reaction conditions: Feed: 0.5 M *o-*xylene in hexane, reaction temperature: 200 °C; H_2_ flow = 25 mLN/min; pressure = 28 bar.

Finally, in order to evaluate the reusability and stability of the supported POM-based catalysts, the effect of reactivating the catalysts on their performance was also investigated. This was achieved by performing a series of 10 reactions at continuously increasing temperatures from 150 to 250 °C, followed by a reactivation according to the initial reduction procedure, then carrying out a second series of 10 reactions under the same conditions. To that purpose, (IrCp*)_4_P_8_W_48_@SBA15 was loaded into the reactor, activated following the standard activation procedure described earlier, allowed to cool down to the reaction temperature and placed on stream starting at 150 °C. The reactions were initiated, and the temperature was increased by 10 °C every 2 h. After 22 h on stream, the system was allowed to cool down and a reactivation step was carried out before repeating the same series of 10 reactions following the same procedure. Interestingly, the conversion of *o-*xylene was reduced by approximately 15% after the reactivation showing some loss in activity. The same observation was made for reactions at 0.10 and 0.15 mLN/min flow rates. The results are summarized in Figure 12.

**Figure 12:**
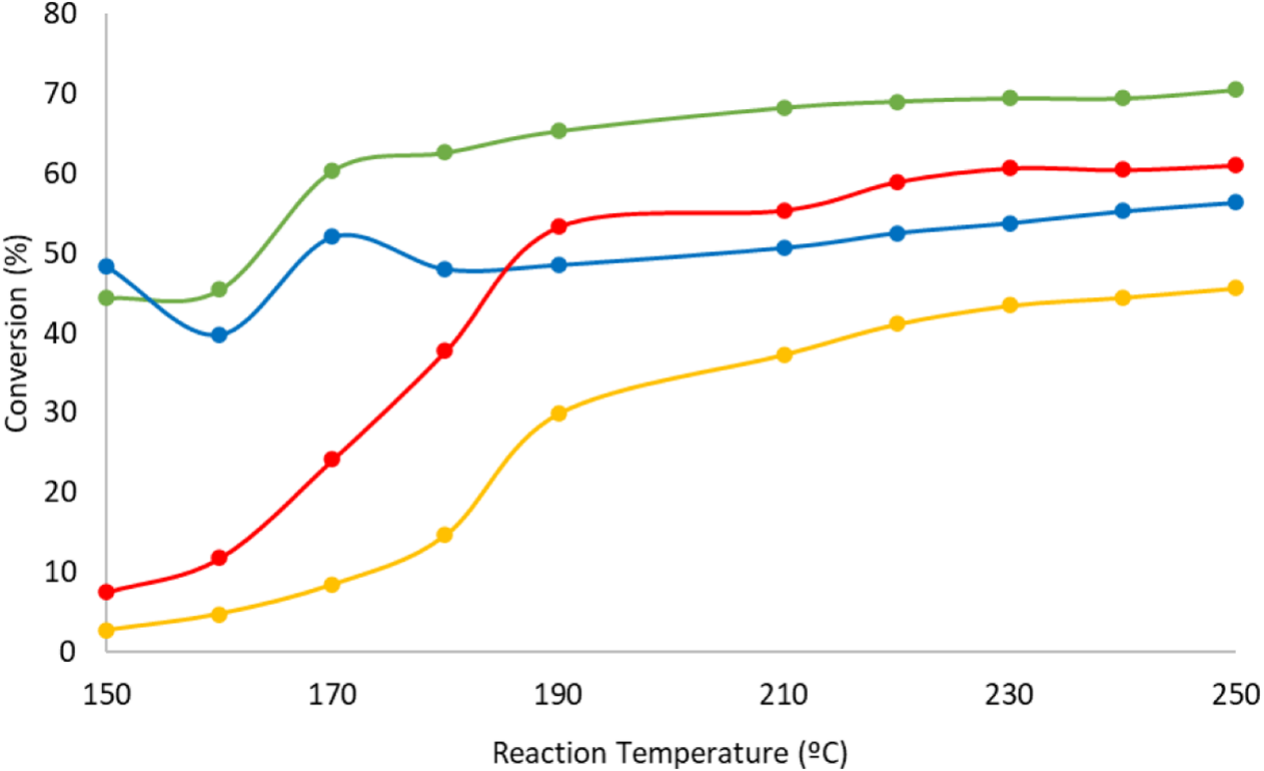
Conversion of *o-*xylene at 0.10 and 0.15 mLN/min flow rates before and after catalyst reactivation. Color code: 0.10 mLN/min before reactivation (green), 0.10 mLN/min after reactivation (blue), 0.15 mLN/min before reactivation (red), and 0.15 mLN/min after reactivation (yellow).

The general concept of Rh- and Ir-Cp*-P_8_W_48_ POMs and their use in hydrogenation was published as WO2021/099142.^49^

Transmission electron microscopy (TEM) studies on **1**@SBA15 and **2**@SBA15 show that precatalysts **1** and **2** are highly dispersed after immobilization on the surface of apts-modified SBA15 (Table 2). Upon calcination, nanoparticles in the range of 0.5–2 nm were observed. After activation and 1 week on stream, the surface of SBA15 is populated with a mixture of 1–2 nm particles and 10–50 nm deposits which can be assigned to agglomerates of W and noble metals in the channels of the support. These results suggest that the stability of **1**@SBA15 and **2**@SBA15 slowly decreases with time under harsh reaction conditions and continuously variable reaction temperatures. TEM images showing the aforementioned agglomeration are displayed in the Supporting Information (Figure S12).

**Table 2:**
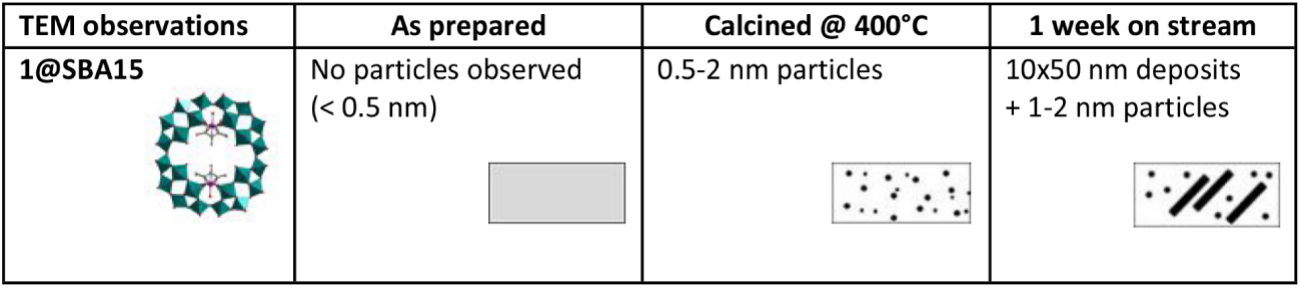
Summary of the TEM Results Performed on **1**@SBA15

The destabilization and agglomeration of the nanoparticles can be partly assigned to the decomposition of the wheel-shaped P_8_W_48_ under the reaction conditions. This was further demonstrated by conducting a thermal stability study on **KLi-P**_**8**_ **W**_**48**_. Figure 13 displays the FTIR of **KLi-P**_**8**_ **W**_**48**_ after heating to different temperatures ranging from room temperature (RT) to 500 °C. These data provide a comprehensive overview of the thermal stability of the crown-shaped polyanion P_8_W_48_. The FTIR spectra of the polyanion at RT to 300 °C suggest that it is stable, but the polyanion starts to become thermally labile upon reaching 400 °C and decomposes at 450 to 500 °C.

**Figure 13:**
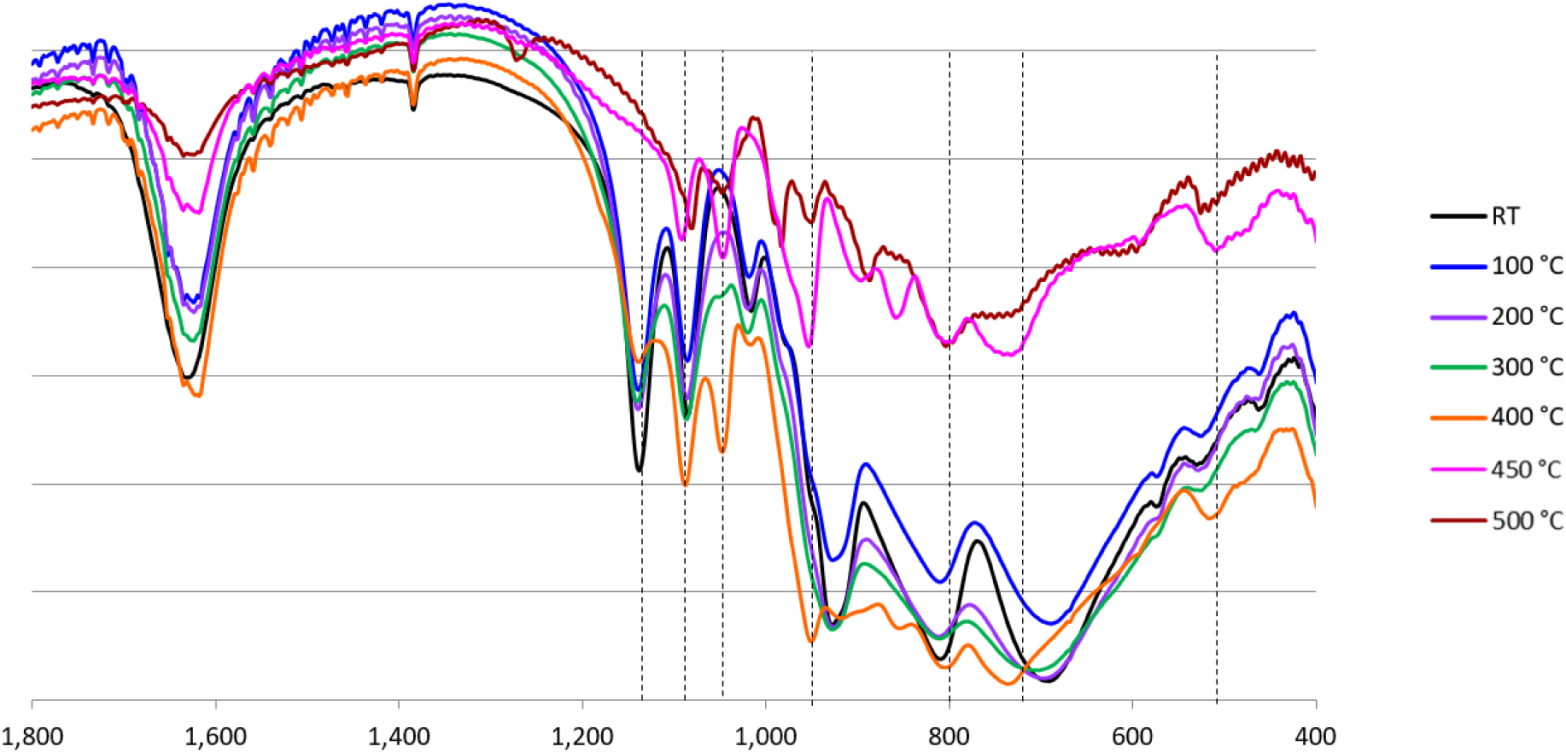
FTIR spectra of **KLi-P_8_W_48_** heated at various temperatures (1 wt % in KBr pellet).

## Conclusions

We have synthesized the first two organometallic rhodium­(III)- and iridium­(III)-substituted polyanions **1** and **2** based on the P_8_W_48_ wheel. The novel polyanions were characterized in the solid state by single-crystal X-ray diffraction, infrared spectroscopy, and thermogravimetric analysis, and in solution by ^31^P and ^13^C NMR spectroscopy. Polyanions **1** and **2** are only the second and third noble-metal derivatives of the wheel-shaped P_8_W_48_. In this work, we have demonstrated that Rh and Ir ions can be grafted inside the P_8_W_48_ host in the form of organo-Rh/Ir precursor complexes. The catalytic activity of the novel polyanions **1** and **2** toward the selective hydrogenation of olefins was also investigated after immobilization of the polyanions on apts-modified SBA15 and characterization using N_2_-adsorption and transmission electron microscopy (TEM). Both precatalysts **1**@SBA15 and **2**@SBA15 exhibited high activities in the hydrogenation of *o-*xylene with different selectivity to *cis-* and *trans*-dimethylcyclohexane well as to ring-opening and cracking products. Furthermore, the effect of reaction temperature, feed flow rate, hydrogen flow rate, and reactivation was investigated. Our work demonstrates the structural diversity in noble metal POM chemistry and its relevance for catalytic applications.

## Supplementary Material



## Notes

FTIR of **1** and **2**, LT ^13^C NMR of **1**, TGA of **1** and **2** and Rh/Ir-precursors, N_2_-adsorption results, details of GC method used for analysis of catalytic reactions (PDF)
