# Supplementary material for: Organorhodium and Iridium-Containing Derivatives of the 48-Tungsto-8-phosphate Wheel: Synthesis, Characterization, and Catalytic Activity
Source: Inorg Chem. 2026 Mar 18;65(12):6682–93. doi: 10.1021/acs.inorgchem.5c06046 (PMC13040535; doi:10.1021/acs.inorgchem.5c06046)
Supplement: Supplementary file 1 [file ic5c06046_si_001.pdf]

Supporting Information

Organorhodium and Iridium-Containing Derivatives of the 48-Tungsto-8-Phosphate Wheel: Synthesis, Characterization and Catalytic Activity

Ali S. Mougharbel,[a] Saurav Bhattacharya,[a,b] Anupam Sarkar,[a] Anton-Jan Bons,[c] Tom D'hondt,[c] Helge Jaensch,[c] and Ulrich Kortz*,[a]

[a] School of Science, Constructor University, Campus Ring 1, 28759 Bremen, Germany. Email ukortz@constructor.university

[b] Department of Chemistry, Birla Institute of Technology and Science, Pilani, KK Birla Goa Campus, Zuarinagar, Sancoale, Goa 403726, India.

[c] ExxonMobil Petroleum & Chemical BV, 1831, Machelen, Belgium.

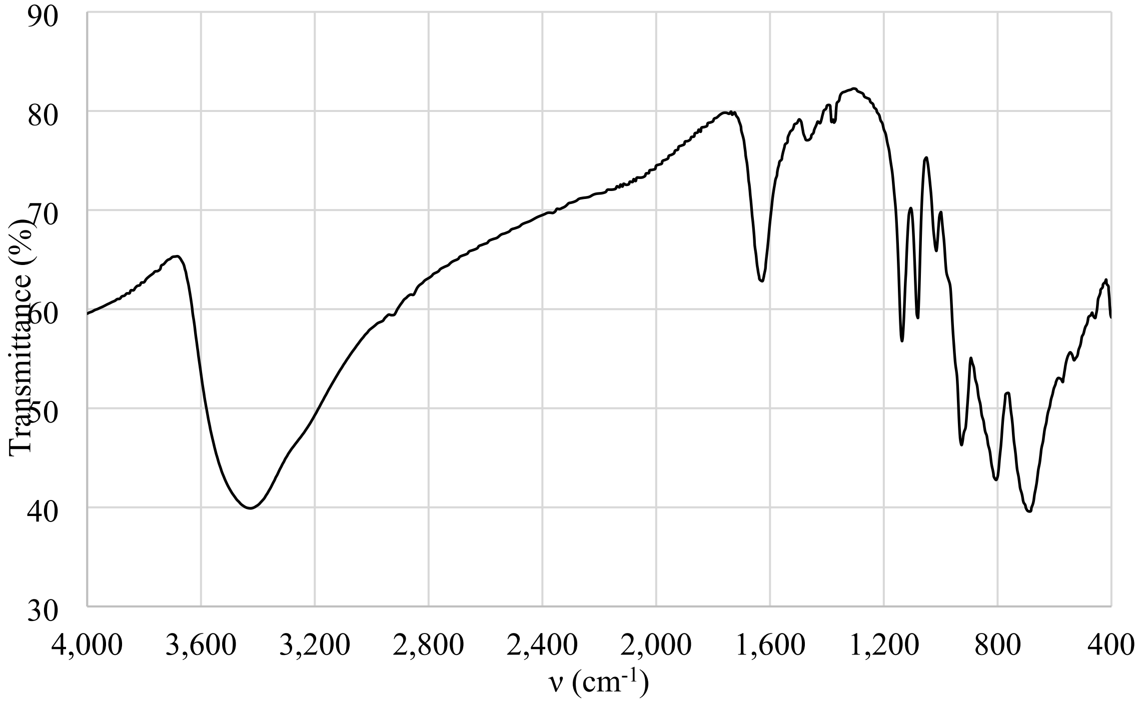

Figure S1. FTIR spectrum of **LiK-1** (1 wt% in KBr pellet).

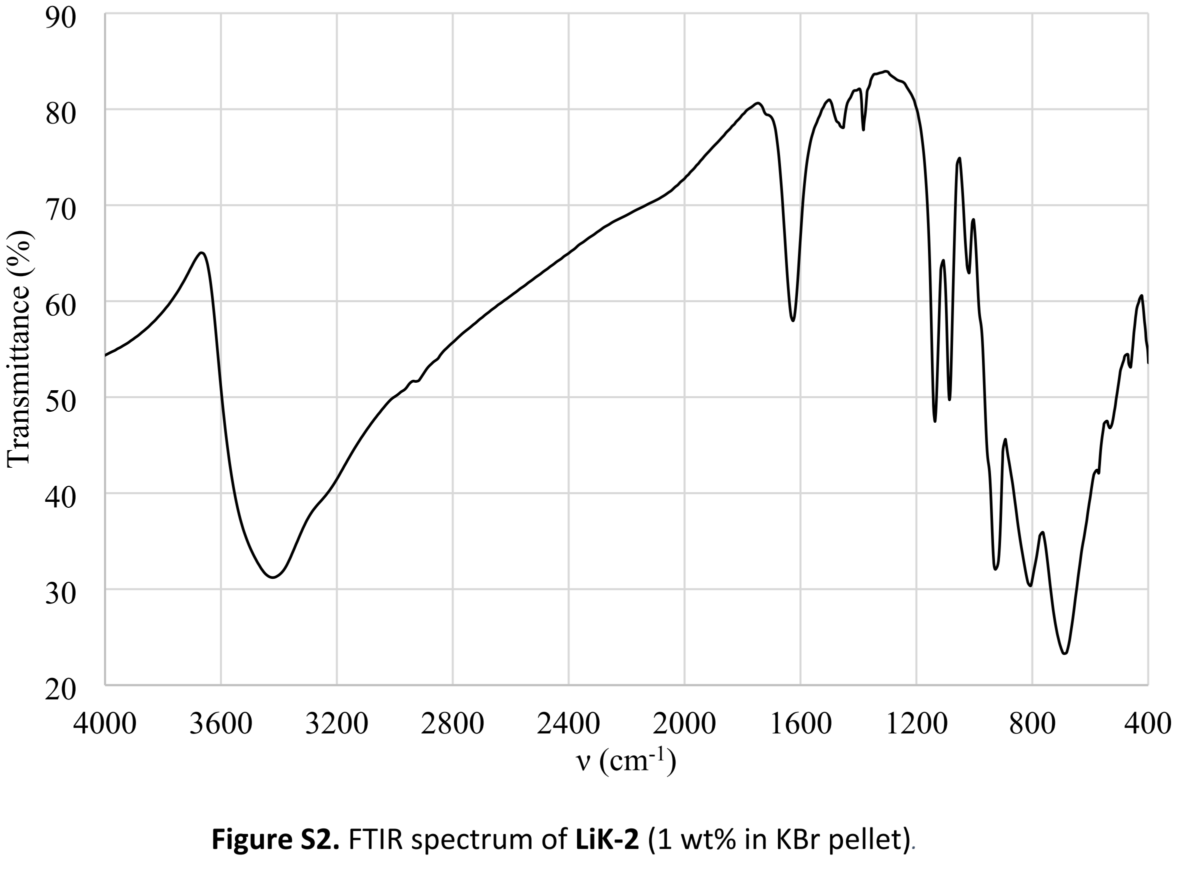

Figure S2. FTIR spectrum of **LiK-2** (1 wt% in KBr pellet).

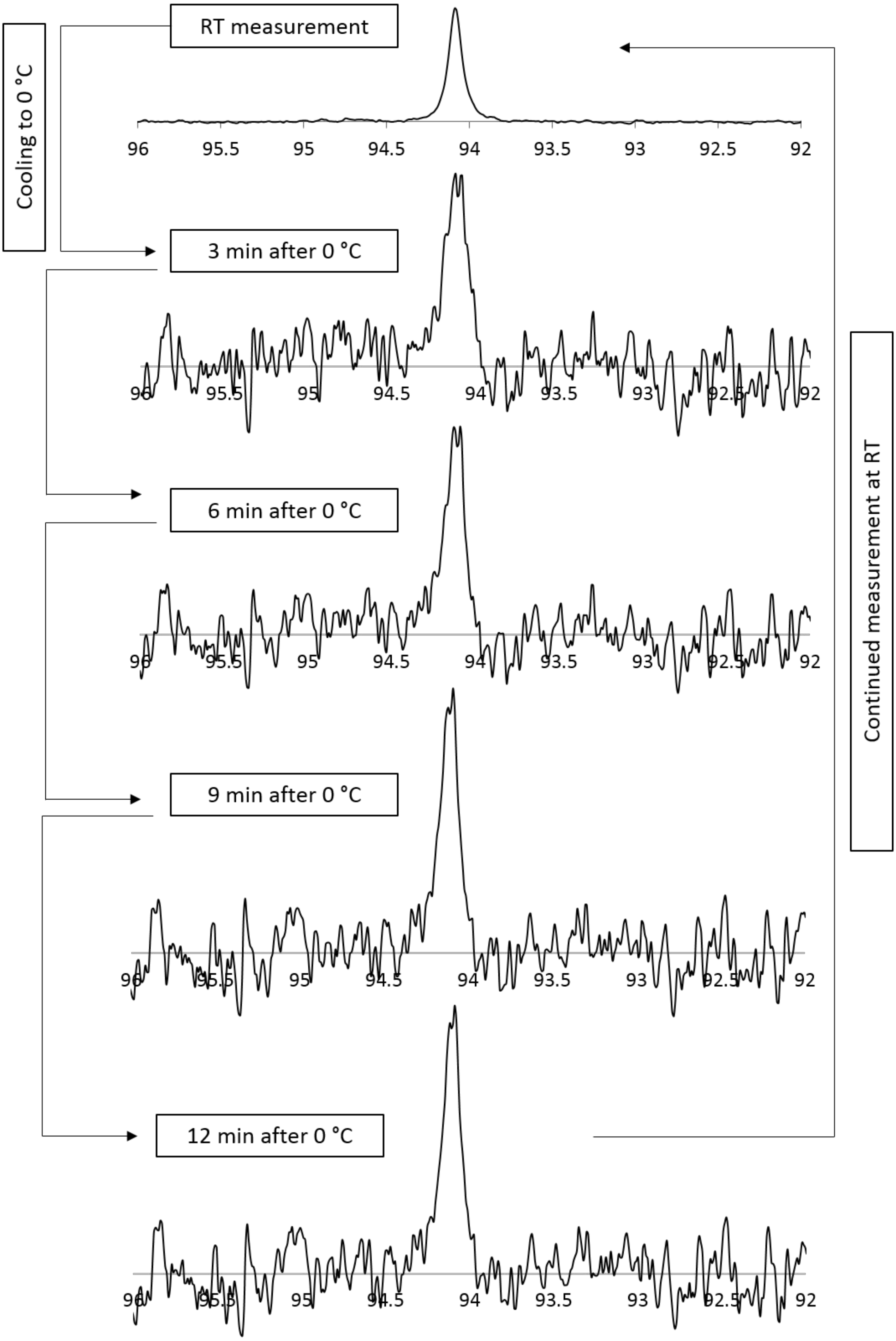

Figure S3. ^{13}C NMR spectra of **LiK-1** in D_2O showing the C-Rh coupling, which is only observed at low-temperature measurement.

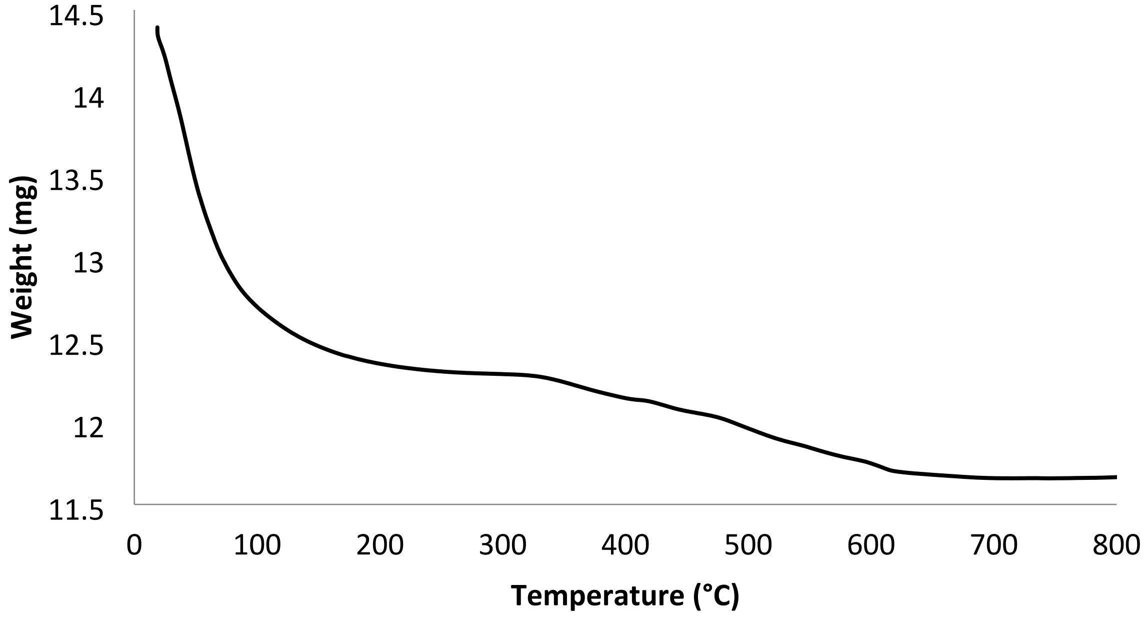

Figure S4. Thermogram of **LiK-1** under N_2 flow of 100 ml/min with temperature ramp of 3 °C/min.

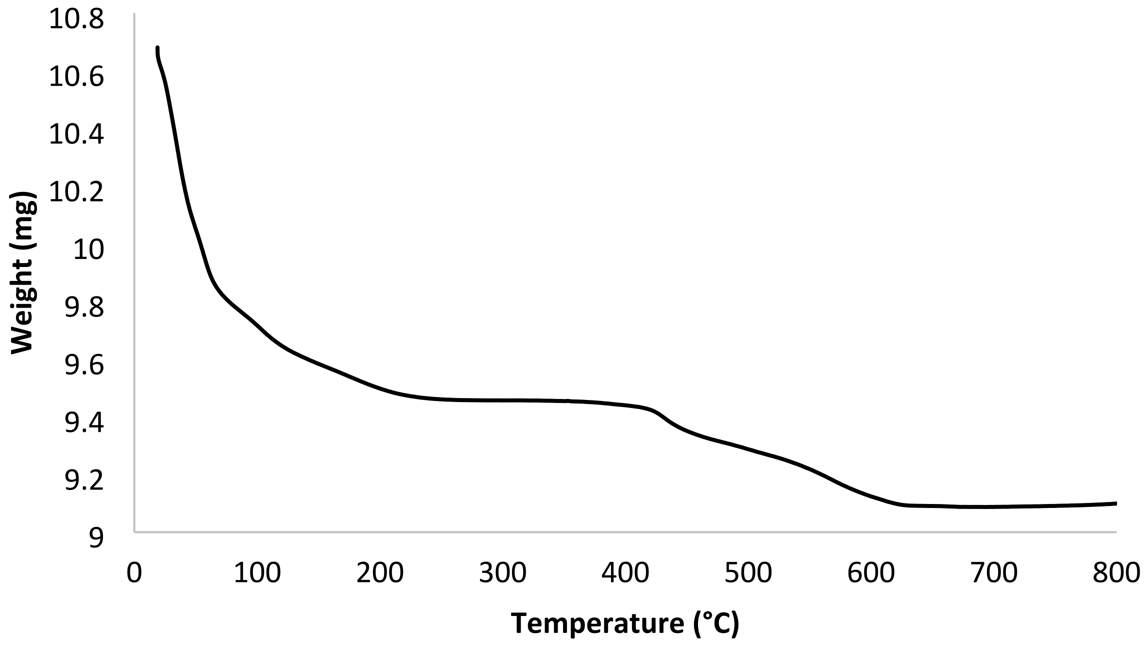

Figure S5. Thermogram of **LiK-2** under N_2 flow of 100 ml/min with temperature ramp of 3 °C/min.

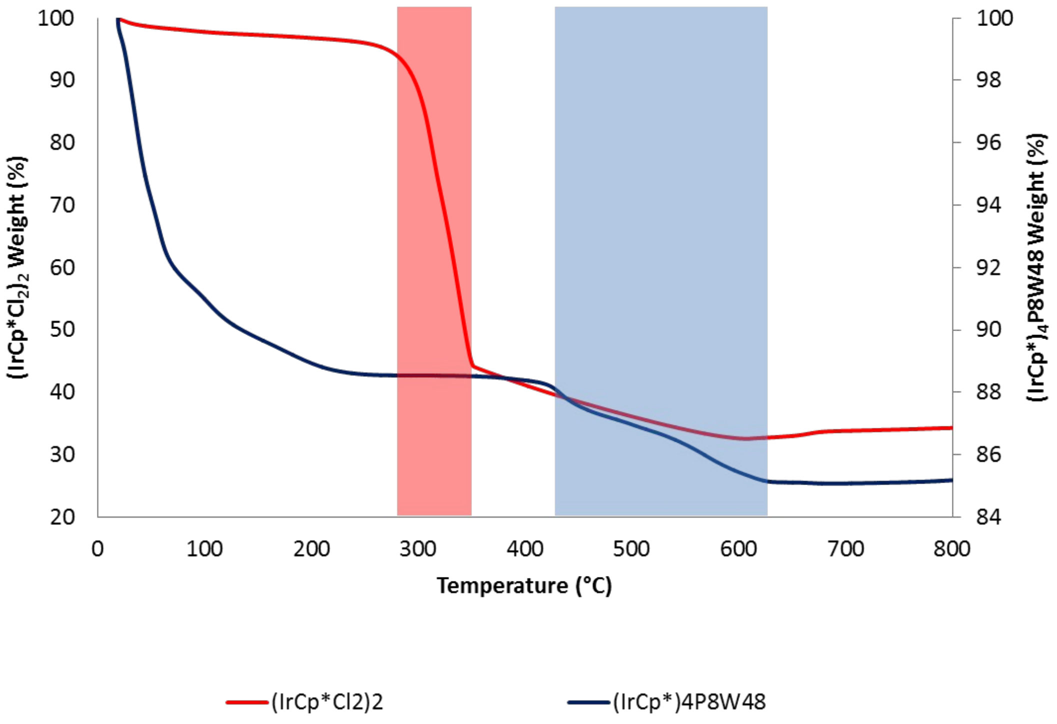

Figure S6. Thermograms showing increasing thermal stability of the IrCp* group before and after coordination to P_8W_{48}. Measurements performed under N_2 flow of 100 ml/min with temperature ramp of 3 °C/min.

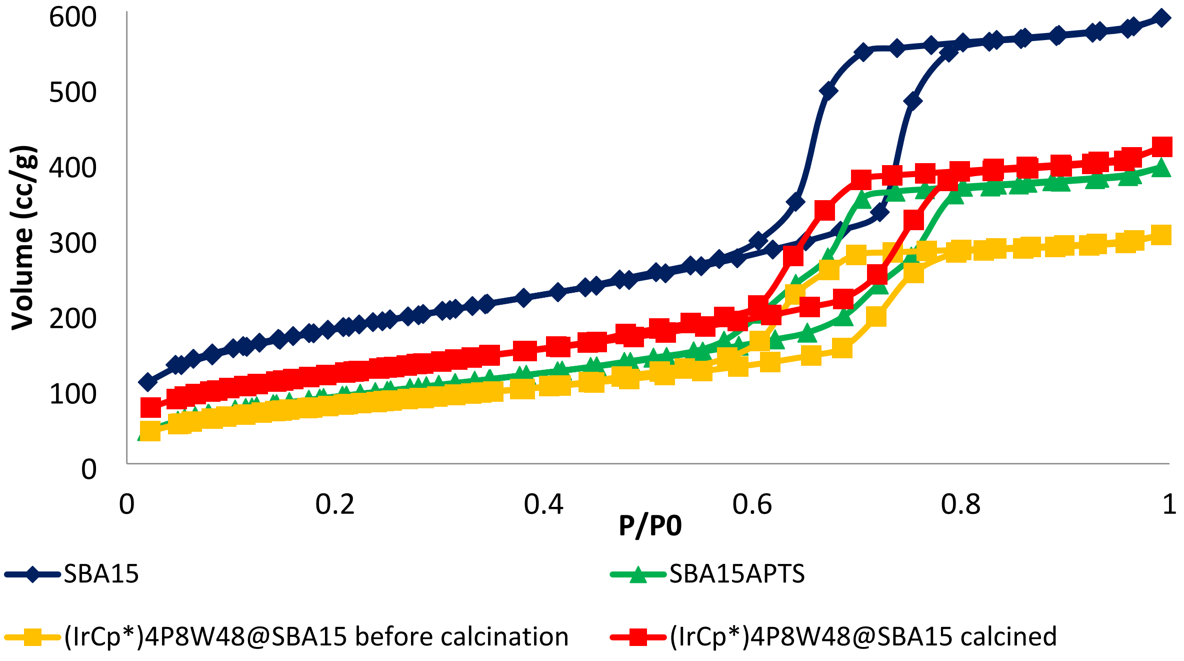

Figure S7. N_2-adsorption isotherms showing the variation in the surface area and pore volume of the support after modification, after immobilization of polyanion **2** and after calcination.

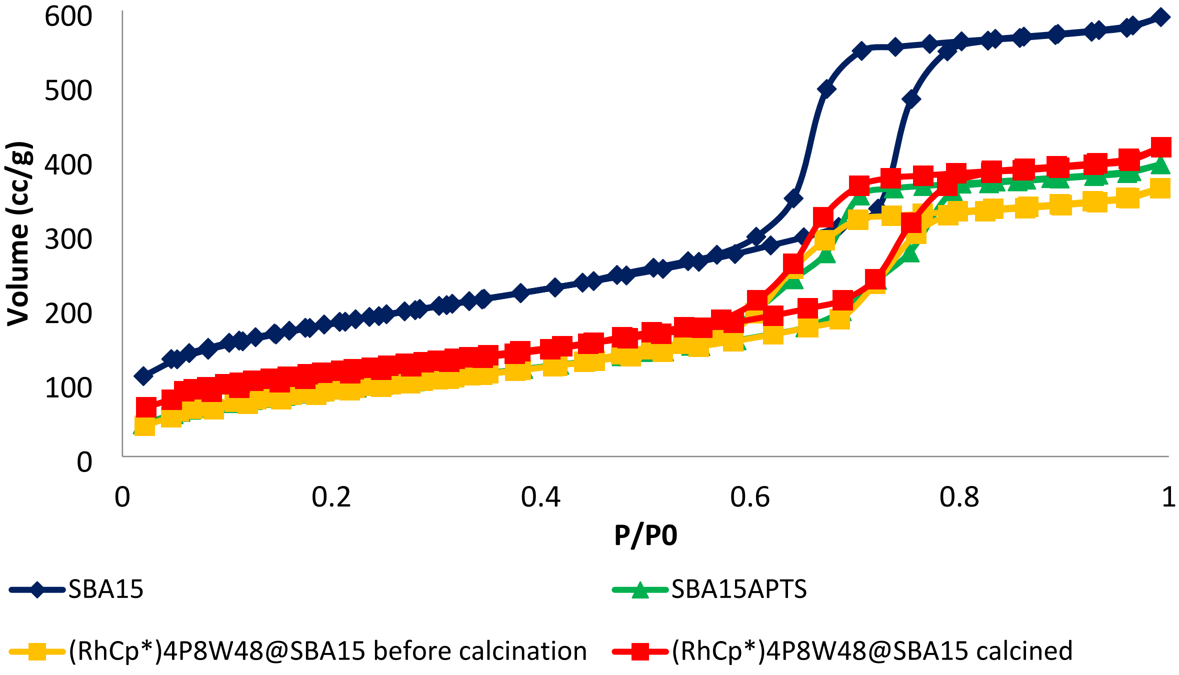

Figure S8. N_2-adsorption isotherms showing the variation in the surface area and pore volume of the support after modification, after immobilization of polyanion **1** and after calcination.

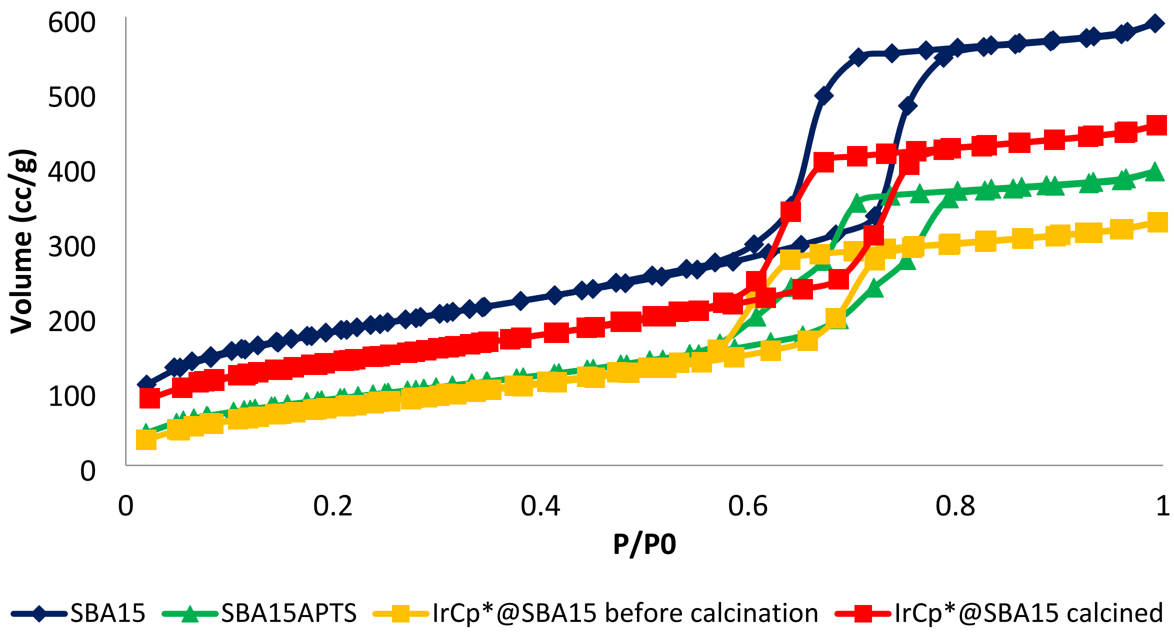

Figure S9. N_2-adsorption isotherms showing the variation in the surface area and pore volume of the support after modification, after immobilization of $(IrCp^*Cl_2)_2$ and after calcination.

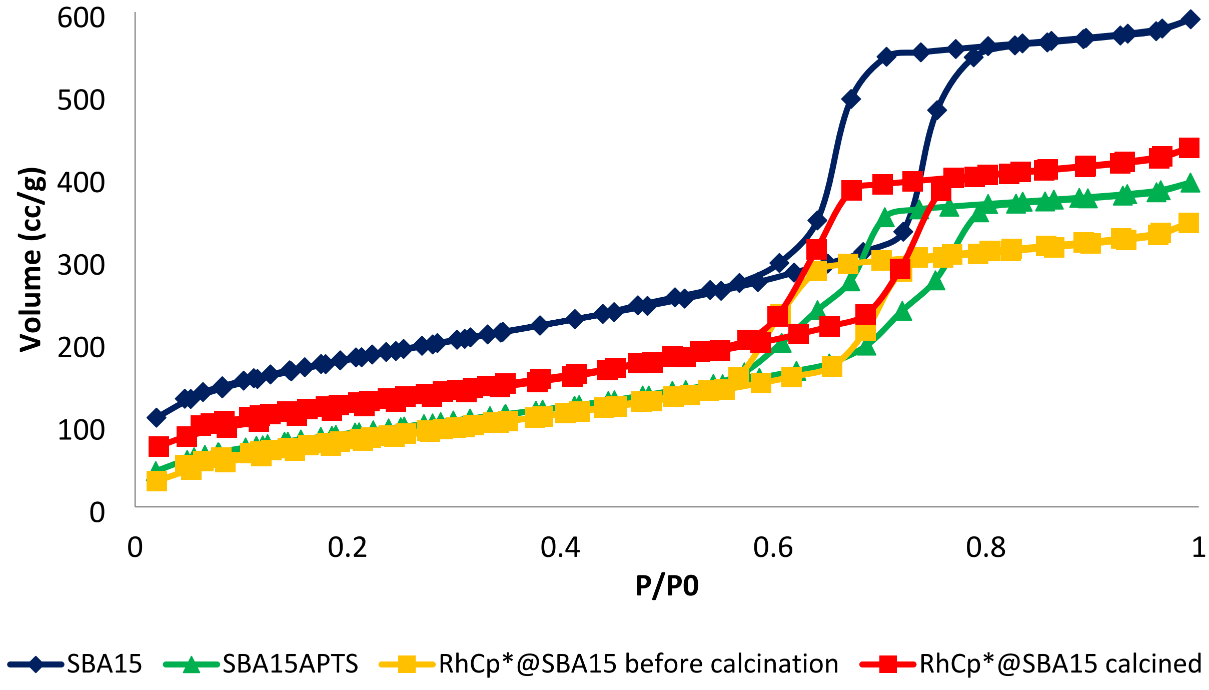

Figure S10. N_2-adsorption isotherms showing the variation in the surface area and pore volume of the support after modification, after immobilization of $(RhCp^*Cl_2)_2$ and after calcination.

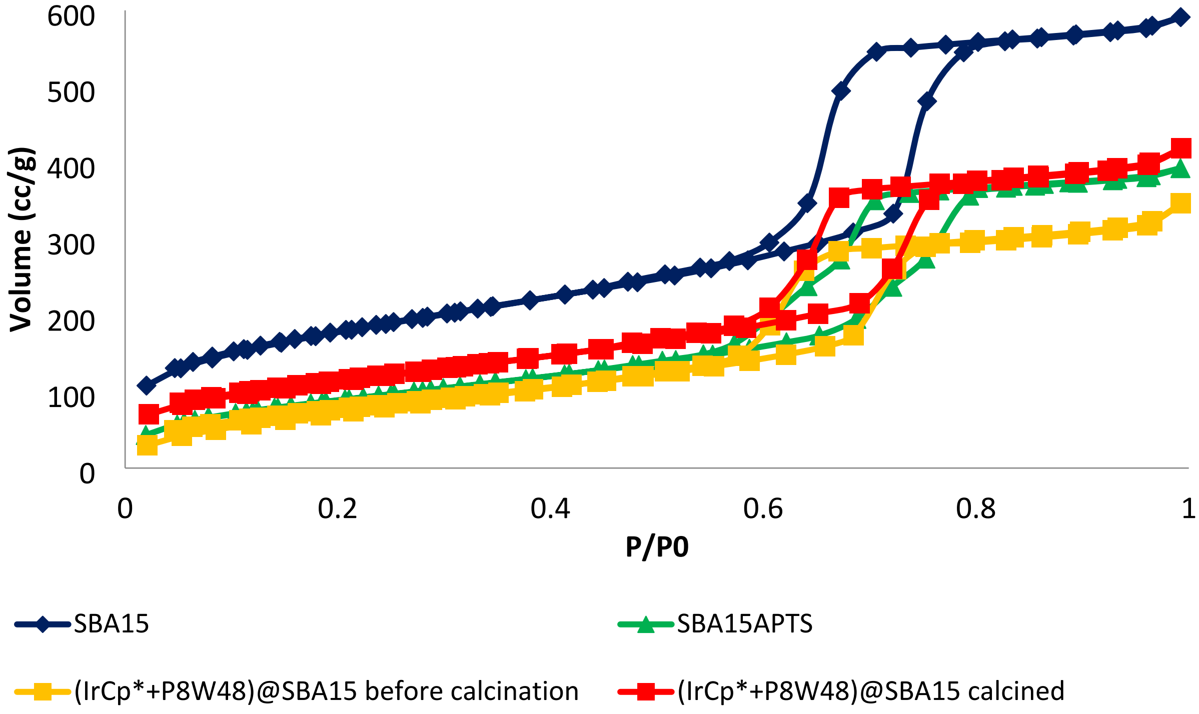

Figure S11. N_2-adsorption isotherms showing the variation in surface area and pore volume of the support after modification, after immobilization of [(IrCp*Cl_2)$_2$ + P_8W_{48}] and after calcination.

Equipment:

Transmission Electron Microscope (TEM): FEI TecnaiG2 F20 ST (10569068) with EDAX EDS spectrometer, operated at 200 kV, single-tilt low-background holder.

Imaging modes:

Bright Field (BF) TEM and High Angle Annular Dark Field Scanning Transmission Electron Microscopy (HAADF-STEM).

Sample preparation:

Samples were crushed in an agate mortar, dispersed in ethanol by sonication, and evaporated on a lacey carbon support foil on a copper TEM grid.

Figure S12. See below for several TEM images of the supported polyanions **1**@SBA15 and **2**@SBA15.

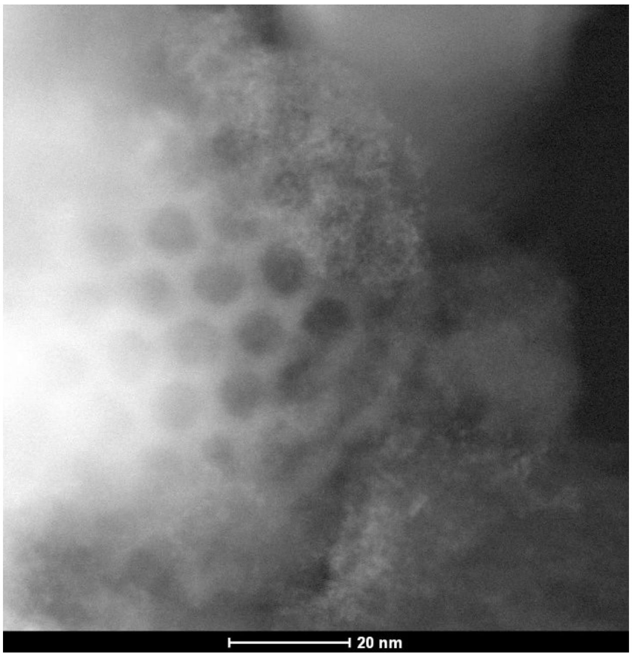

HAADF-STEM of as synthesized **2**@SBA15.

1-2 nm metal particles, mostly in clusters on the external surface of the SBA15.

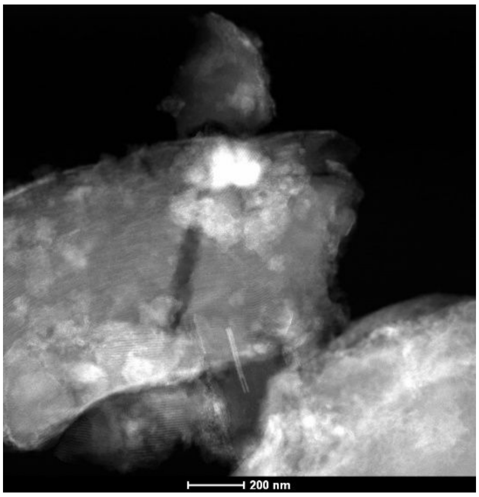

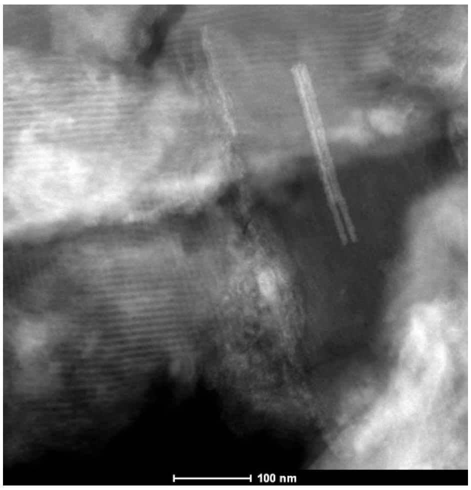

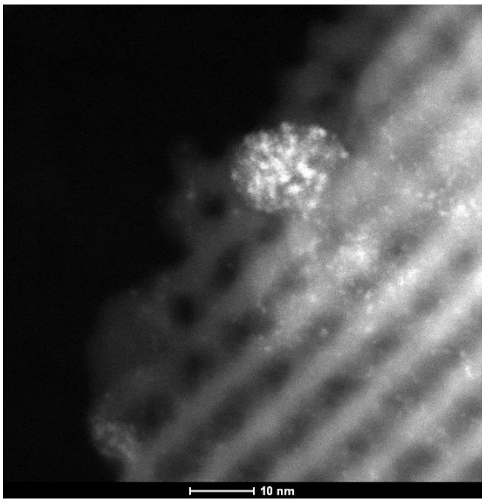

HAADF-STEM of **2**@SBA15 after calcination (400 °C, 4 hours, 0.5 °C/min ramp).

1-2 nm metal particles, clusters, ribbons, strings, and larger particles.

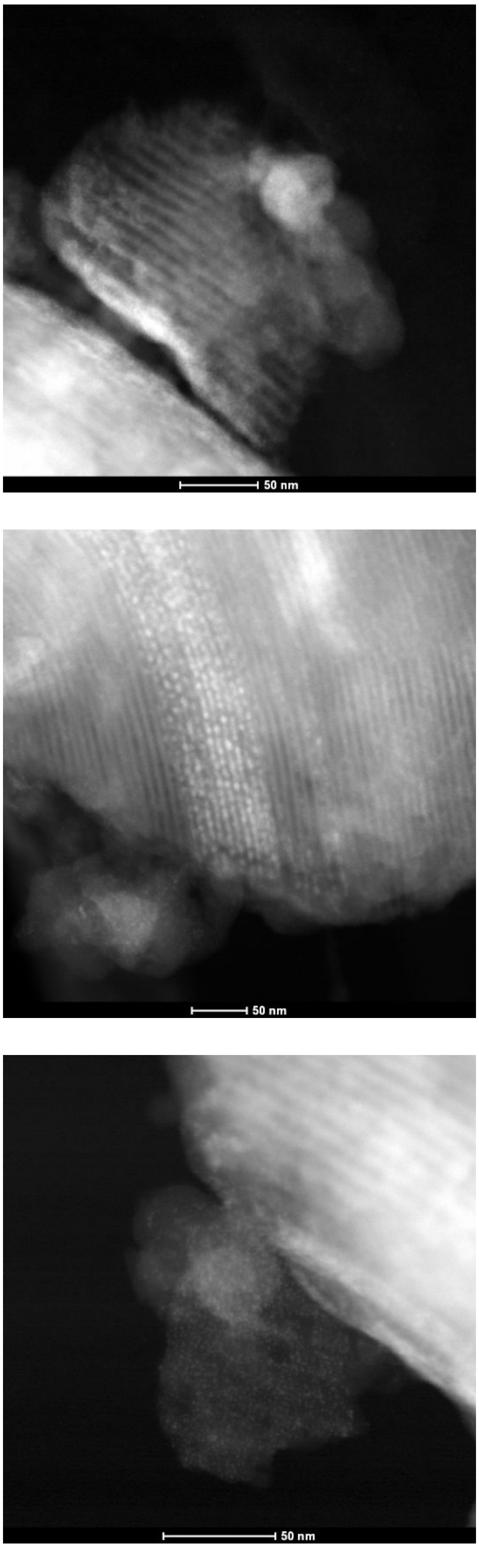

HAADF-STEM of **2**@SBA15 after reaction (one week on stream).

Larger particles as well as 1-2 nm metal particles on outer surface of SBA15 support, strings of particles aligned with SBA15 channels. Element maps show that metal deposited in the channels is predominantly W with some Ir.

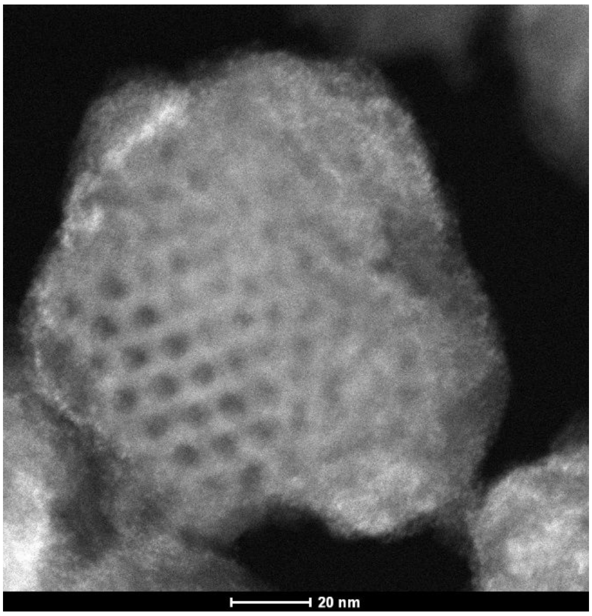

HAADF-STEM of as synthesized **1**@SBA15.

1-2 nm metal particles, mostly in clusters on the external surface of SBA15.

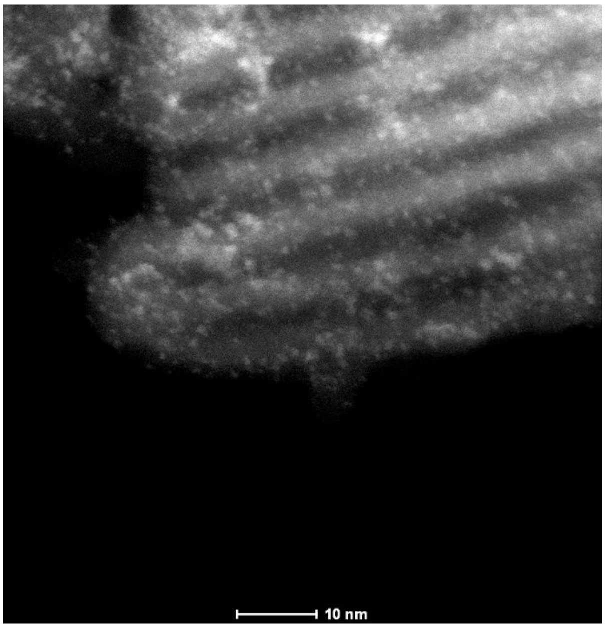

HAADF-STEM of **1**@SBA15 after calcination (400 °C, 4 hours, 0.5 °C/min ramp).

1-2 nm metal particles.

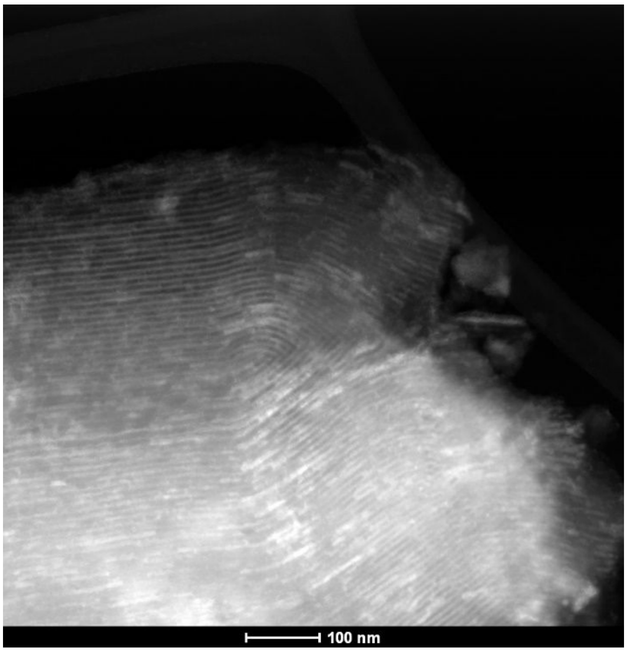

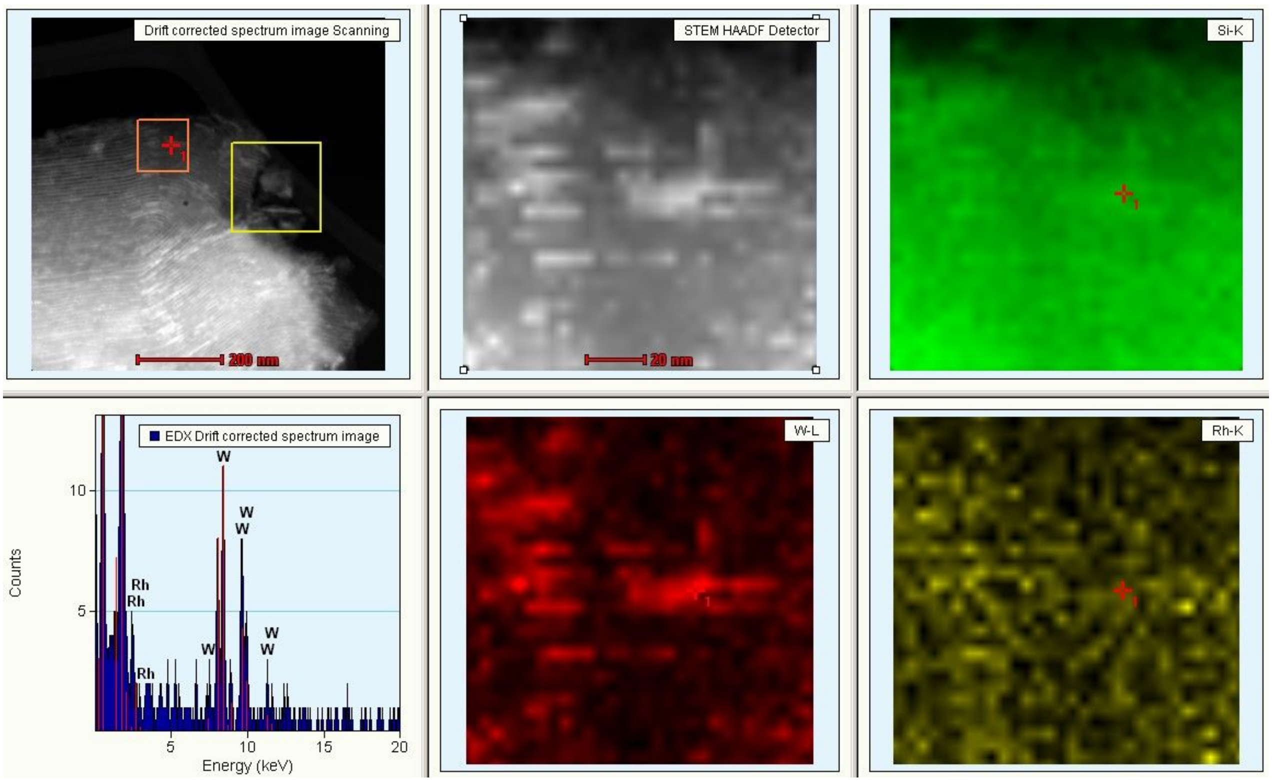

1@SBA15 after reaction (one week on stream).

Imaging suggests metal deposition as particles on surface and in the channels.

Table S1. Variation of surface area and pore size of the prepared materials after modification, POM immobilization and calcination.

	Surface Area (m2/g)	*Pore Volume (cc/g)*
SBA15	652	0.92
SBA15-APTS	334	0.61
2@SBA15 before calcination	287	0.47
2@SBA15 after calcination	433	0.65
1@SBA15 before calcination	330	0.56
1@SBA15 after calcination	412	0.64
IrCp*@SBA15 before calcination	292	0.50
IrCp*@SBA15 calcined	501	0.70
RhCp*@SBA15 before calcination	302	0.53
RhCp*@SBA15 calcined	451	0.67
(IrCp*+P_8W_{48})@SBA15 before calcination	287	0.54
(IrCp*+P_8W_{48})@SBA15 calcined	416	0.65

GC-FID Method parameters:

Inlet settings:
Temperature: 300 ˚C
Mode: Split
Pressure: 3.14 bar

Oven settings:

Oven	Rate °C/min	Value °C	Hold Time min	Run Time min
(Initial)		35	15	15
Ramp 1	1	60	20	60
Ramp 2	2	200	0	130

Post run temperature: 250 ˚C
Post run time: 5 min

Detector settings:
Temperature: 280 ˚C
H_2 flow: 45 ml/min
Air flow: 450 ml/min
Make up flow (N_2): 1 ml/min

Column settings:
Pressure: 3.14 bar
Flow: 2.2 ml/min
